# Exploration of Multi-State Conformational Dynamics and Underlying Global Functional Landscape of Maltose Binding Protein

**DOI:** 10.1371/journal.pcbi.1002471

**Published:** 2012-04-19

**Authors:** Yong Wang, Chun Tang, Erkang Wang, Jin Wang

**Affiliations:** 1 State Key Laboratory of Electroanalytical Chemistry, Changchun Institute of Applied Chemistry, Chinese Academy of Sciences, Changchun, Jilin, China; 2 State Key Laboratory of Magnetic Resonance and Atomic and Molecular Physics, Wuhan Institute of Physics and Mathematics, Chinese Academy of Sciences, Wuhan, Hubei, China; 3 College of Physics, Jilin University, Changchun, Jilin, China; 4 Department of Chemistry, Physics and Applied Mathematics, State University of New York at Stony Brook, Stony Brook, New York, United States of America; University of California, Irvine, United States of America

## Abstract

An increasing number of biological machines have been revealed to have more than two macroscopic states. Quantifying the underlying multiple-basin functional landscape is essential for understanding their functions. However, the present models seem to be insufficient to describe such multiple-state systems. To meet this challenge, we have developed a coarse grained triple-basin structure-based model with implicit ligand. Based on our model, the constructed functional landscape is sufficiently sampled by the brute-force molecular dynamics simulation. We explored maltose-binding protein (MBP) which undergoes large-scale domain motion between open, apo-closed (partially closed) and holo-closed (fully closed) states responding to ligand binding. We revealed an underlying mechanism whereby major induced fit and minor population shift pathways co-exist by quantitative flux analysis. We found that the hinge regions play an important role in the functional dynamics as well as that increases in its flexibility promote population shifts. This finding provides a theoretical explanation of the mechanistic discrepancies in PBP protein family. We also found a functional “backtracking” behavior that favors conformational change. We further explored the underlying folding landscape in response to ligand binding. Consistent with earlier experimental findings, the presence of ligand increases the cooperativity and stability of MBP. This work provides the first study to explore the folding dynamics and functional dynamics under the same theoretical framework using our triple-basin functional model.

## Author Summary

A central goal of biology is to understand the function of the organism and its constituent parts at each of its scales of complexity. Function at the molecular level is often realized by changes in conformation. Unfortunately, experimental explorations of global motions critical for functional conformational changes are still challenging. In the present work, we developed a coarse grained triple-well structure-based model to explore the underlying functional landscape of maltose-binding protein (MBP). By quantitative flux analysis, we uncover the underlying mechanism by which the major induced fit and minor population shift pathways co-exist. Though we have previously lent credence to the assertion that dynamical equilibrium between open and minor closed conformations exist for all the free PBPs, the generality of this rule is still a matter of open debate. We found that the hinge flexibility is favorable to population shift mechanism. This finding provides a theoretical explanation of the mechanism discrepancies in PBP protein family. We also simulated the folding dynamics using this functional multi-basin model which successfully reproduced earlier protein melting experiment. This represents an exciting opportunity to characterize the interplay between folding and function, which is a long-standing question in the community. The theoretical approach employed in this study is general and can be applied to other systems.

## Introduction

Biomolecular function is executed through conformational dynamics at physiological conditions, ranging from small fluctuations in atomic positions to large movements of parts of or even entire molecules [1], [2]. It has been recognized that large-scale domain rearrangement is involved in processes like protein folding, molecular recognition, enzyme catalysis, signal transduction, transcriptional regulation and allostery, and plays an important role in biomolecular machines, such as the ribosome, transporter, molecular chaperones, enzyme, and molecular motors, carrying out their respective functions [3].

Though local conformational changes can be detected using experimental probes such as NMR, there are still a number of challenges for exploring large conformational changes in experiment. On the other hand, computational exploration of large conformational changes is made difficult by the fact that the time scale of the conformational changes are often on the order of sub-seconds to minutes, while the molecular dynamics simulations for moderately sized proteins are on the order of microseconds, thus presenting a challenge for modeling conformational changes. Advancing the modeling in this would not only provide us the local information and connect the modeling directly to experiments, but would also predict and give guidance for the ongoing and future experimental explorations of global conformational changes.

In recent years, theoretical models have been developed to explore functional dynamics near the bottom of the energy landscape through integrating native structural information from two reference states. These models have been used to investigate the conformational transitions of typical allosteric proteins such as adenylate kinase (ADK) [4]–[8], Rop dimer [9], GFP [10], glutamine-binding protein [11], Arc repressor [12], calmodulin [13], [14], Src kinase [15], NtrC [16] and protein kinase A [17] etc. With advanced experimental approaches, more and more systems have been revealed to have more than two states. While it is well established that the inactive ligand-free state and the active ligand-bound state are both critical to protein function, it is worth emphasizing that other metastable conformational states are also of importance. However, the present models with two basins seem to be insufficient to describe such multiple-state systems. Recently, a multiple-basin landscape was explored from microscopic models [5] and macroscopic models [18], [19]. In these models either the realization of macroscopic states is hard to control or the implementation is not readily available in common molecular modeling packages [19]. In this work, we developed a coarse grained triple-well structure-based model with the consideration of ligand binding, extending the two-state approach [7] for recapitulating multiple state large amplitude conformational changes.

We explored maltose binding protein (MBP) which has three observed states. MBP is a member of the large family of periplasmic binding proteins (PBPs) [20] with a similar (two-domain) folding structure and size, linked by a rather flexible 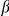-strand region. Proteins in PBPs family display a common functional conformational switch between the apo-open state and the holo-closed state upon ligand binding. This is proposed to be a consequence of natural selection for fitting their function for efficient uptake in the periplasm and for directed chemotaxis [21]. A database of the PBP superfamily with hundreds of X-ray structures from E. coli, thermophilic bacteria and eukaryotes etc., is a treasure trove for studying the relationship between ligand binding and conformational coupling as well as for applications in protein engineering [20].

MBP is found in the periplasmic space of gram-negative bacteria and serves as a receptor for osmotic shock and chemotaxis in response to the maltose and other maltodextrins from the environment [22], [23]. It has been extensively studied by X-ray crystallography [24]–[27], NMR spectroscopy [28]–[30], atomic force microscope [31] and other biophysical techniques [32], [33]. Recently, NMR paramagnetic relaxation enhancement (PRE) studies [33] have revealed that a minor species of apo MBP which represents a partially closed state in solution without ligands. This is in addition to the major species and is consistent with the structure of open MBP resolved by X-ray. Presently, we face several mechanistic questions: What are the processes by which conformations change? Is the system better described by an induced-fit [3] or population shift mechanism [34]? How many reaction pathways are there? What are their importance and the relative weights? Which part(s) of the protein plays the most important role? We addressed these questions using molecular dynamics simulations based on our structure based model.

So far, there are relatively few computational studies on the conformational dynamics of MBP [35]–[38]. Stockner et al. used short-time (30 ns) atomic MD simulation to investigate the functionally important transition of MBP [35]. They observed an open-to-closed transition starting from the open structure with ligand and a closed-to-open transition from close structure without ligand. However, the minor partially closed state was not found during the process, reflecting that the simulation time scale is insufficient. Most recently, the dynamical equilibrium between open and apo-closed states in free MBP was validated in MD simulations carried out by Bucher et al. [36], [37] and Kondo et al. [38]. They explored the conformational dynamics by an enhanced sampling algorithm called accelerated MD [39], or by umbrella sampling to overcome the high free energy barriers. These methods both require one to modify the true potential by introducing a biasing potential into the system, making it difficult to obtain kinetic information. It is still challenging to draw definitive conclusions due to the computational bottleneck in the number of transition events.

**Figure 1:**
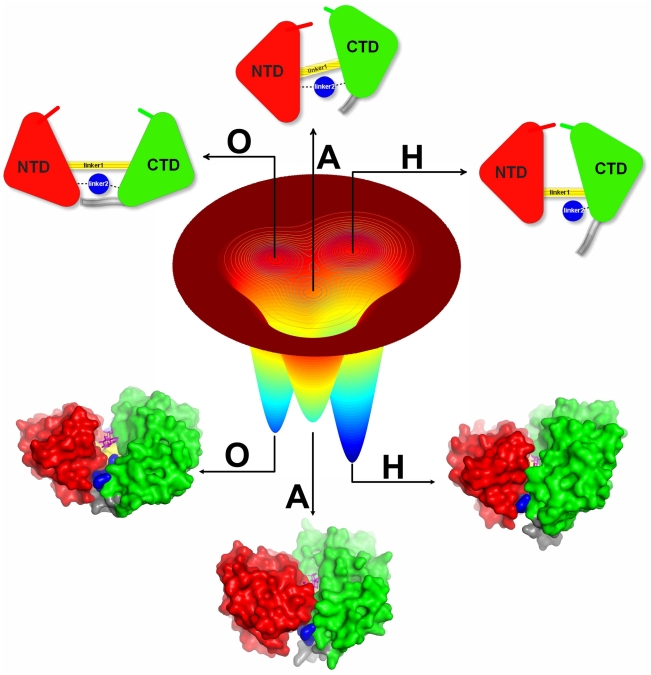
Schematic diagram of triple-basin landscape constructed by integrating structural information from open (O), apo-closed (A) and holo-closed (H) states of MBP. MBP contains two globular domains, NTD (colored in red) and CTD (colored in green) which can be further divided into C1 domain and C2 domain, sharing similar secondary structures and topology with a central 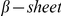 and on both sides with two or three parallel 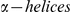. These two large globular domains move back and forth by two linkers: linker1 (colored in yellow) and linker2 (colored in blue). The ligand-binding site is located at the base of a deep groove formed between the two domains. This functional interface, called the “balancing interface”, was considered to play a role as a “molecular switch” that triggers the conformational turnover. In fact, the balancing interface includes two important segments. One is a loop region (residues Y167 to D173, colored in grey) as a part of C2 domain, As MBP in open state, this loop interacts with NTD to form a number of contacts which are broken in holo-closed state or apo-closed state. Another is the linker2 (residues D310 to P330, colored in blue) which plays important roles not only in bridging the two domains, but also as the base of the active site groove. In this study, we refer to the loop as the “balancing loop” and the linker2 as the “balancing linker”.

In this work, we explored the multi-state dynamics from a global perspective by building a coarse-grained functional landscape based on available structural information (Figure 1), which was sampled sufficiently by molecular dynamics simulation. The present work allows us to perform a complete dynamical simulation of conformational transitions between all three observed states of MBP, and obtain thousands of transitions giving statistical reliability. In addition, this enables us to extract the thermodynamics and kinetic information directly from the equilibrium MD trajectories. Furthermore, these trajectories can be projected onto any arbitrary coordinates, such as in Cartesian, dihedral, or contact space as suggested in Ref. [40]. This feature is lack in the umbrella sampling approach or similar methods which require prior knowledge of reaction coordinates [39]. Overall, coarse grained models can provide a useful first order approximation and global landscape while the atomistic simulations explore the system in greater detail. The combination of both methods may provide a fuller picture of functional conformational transition. This combination strategy has been applied by us on binding-folding dynamics [41].

Based on our model, we approximated various perturbations including ligand concentration and hinge flexibility. We revealed the dependence of shape of the functional landscape and the robustness of the underlying mechanism of conformational transition in MBP. We established a link between the underlying energy landscape and thermodynamic stability, kinetic paths/speed, and structure function correlation.

## Results

In this work we integrated structural information of three states of MBP to explore the conformational transition mechanisms. The key to construct a triple-basin model is to define a mixed contact map which integrates heterogeneous sources of structural information together. In our model, the mixed contact map contains the core contacts which are shared with all the native states as well as the state-specific contacts. The strengths of the core contacts and the state-specific contacts are rescaled by their energetic contributions which are denoted as 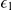 and 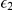, respectively. In addition, the effect of ligand binding is realized by introduction of ligand mediated interactions, which are represented by inter-domain ligand-mediated contacts formed in holo-closed state of MBP. The strength of ligand binding contacts is rescaled by 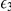. See section of Model and Methods in *Text S1* for more details. In addition, molecular dynamics simulations were used to probe the sensitivity of the model on energetic parameters. Several thousands of basin transitions indicated sufficient sampling.

Recently, a statistical survey for a set of allosteric proteins [42] revealed the robust allosteric feature that the state-specific contacts are significantly weaker than the shared contacts. This feature is likely incorporated to facilitate conformational change by switching only the state-specific contacts. Considering this, the strength of state-specific contacts 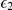 is 0.4 and the strength of shared contacts 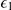 is 1.0. These parameters yield a ratio of state-specific contacts to shared contacts of 0.4. We found that the model worked well as the ratio was between 0.4 and 0.6. This indicates that the allosteric feature found in other allosteric proteins is also shared with MBP. To our knowledge, this work is the first explicit application of this allosteric feature to guide the parametrization of multiple-basin conformational change model.

### Ligand-unbound functional landscapes

Based on dihedral angles parameterized from open state (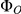 model, see section of Superior Angle Models in *Text S1* for details), we calibrated our triple-well model so that the relative population of open and apo-closed states is comparable to experimental measurements in the absence of ligand binding (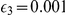).

The free energy surfaces 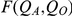 is shown in the two-dimensional space of 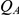 and 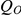 for the ligand-unbound case (Figure S4 in *Text S1*). Here, 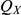 and 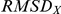 (X can be O, A and H) are the order parameters which are employed to monitor the closeness to their respective states including open, apo-closed and holo-closed states (See the definitions in *Text S1*). (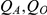) = (0.9, 0.3) for the apo-closed conformation and (0.1, 0.9) for the open conformation. The free energy barrier from apo-closed to open state is 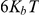, and from open to apo-closed state is 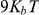. Free energies are correlated with the equilibrium probabilities of the specific states which can be probed in biophysical tools such as spectroscopy, single molecule fluorescence [43], and atomic force microscopy (AFM) [44]. Equilibrium denaturation experiments [30] revealed a linear correlation between free energy of unfolding and the rotation angle between the two domains of MBP. The stability of the (apo)protein decreases with domain closure by about 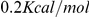 per degree of rotation. By measuring the difference in mechanical work between the ligand-free and ligand-bound states using AFM, the open-closed transition energy for MBPs was determined to be 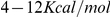 [44]. From our simulation, we can conclude that, for free MBP, the free energy barrier from open state to holo-closed state should be larger than 10 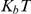, which is consistent with the experiment and provides the physical basis of the underlying energy landscape. We quantify the population of open and apo-closed state to be 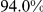 and 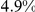, respectively, which agrees well with the paramagnetic relaxation enhancement data. Note that there is about 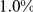 misfolded conformation located outside the native basins from the simulation.

### Effects of ligand binding

**Figure 2:**
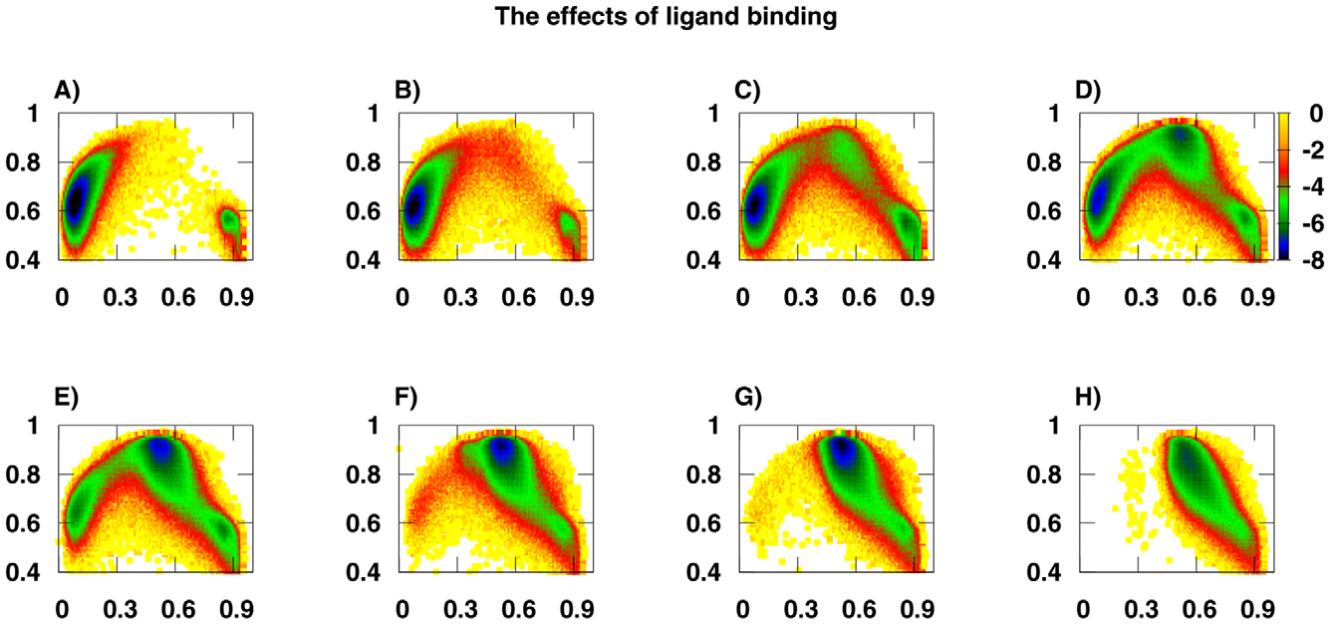
The effects of ligand binding on thermodynamics by increasing 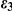
 **from 0.0 to 1.5.** The other parameters are 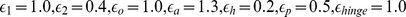. The free energy surfaces 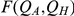 are shown in the two-dimensional space of 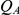 and 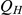 for corresponding 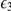 values. (A) 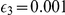, there are two free energy minima. One corresponds to open state at (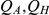) = (0.1,0.6), the other corresponds to apo-closed state state at (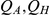) = (0.9,0.6). (B–E) 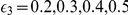, there is a free energy minimum corresponding to holo-closed state at (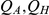) = (0.5,0.9) connects open basin and apo-closed basin. (F–H) 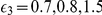, the other two basins gradually disappear as the concentration of the ligands becomes high enough, making the holo-closed basin the unique one.

Next, we investigate the effects of ligand binding by increasing 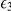 from 0.0 to 1.5. In Figure 2, the free energy surfaces 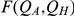 for different ligand-bound ratios are shown in the order parameter space of 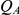 and 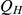. In Figure 2A, there are two free-energy minima: one corresponds to the open state at (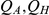) = (0.1,0.6), the other corresponds to the apo-closed state state at (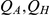) = (0.9,0.6). It indicates that conformational transitions occur between the open state and the apo-closed state in the absence of ligand (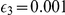). In Figure 2B–E, a free-energy minimum corresponding to the holo-closed state at (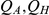) = (0.5,0.9) connects the open basin and the apo-closed basin. This minimum does not exist in the absence of a ligand as shown in Figure 2A and the population of conformations at this minimum is small when binding to ligand with low concentration. In Figure 2F–H, we can see that the two basins for the open and the apo-closed states gradually disappear as the concentration of the ligands becomes high enough, making the holo-closed basin the unique one meaning that the protein stabilizes at the holo-closed state when saturated with bound ligand.

### Induced fit pathways in free energy profiles

We further examine the underlying mechanism of how ligand binding and conformational change of MBP from the open state to the ligand-bound state are coupled. Two distinct mechanisms have been suggested to describe the conformational transition in biomolecular recognition. The first is called induced fit [3]. In this mechanism, the relationship between ligand binding and conformational change is accounted for as ligand binding drives a ligand-free, or apo (usually open) enzyme towards activated conformation. The second proposed mechanism is called conformational selection or population shift [34]. Within this paradigm, the unbound protein takes on multiple native conformations, subsequently, binding to ligand stabilizes the pre-existing higher energy conformation.

For the conformational change of MBP responding to ligand binding, the population shift mechanism indicates that ligand binding stabilizes the pre-existing partially closed state of MBP (A basin), and the protein changes its conformation into fully closed state (H basin) passing through A basin. The induced fit mechanism indicates the direct transitions from O basin to H basin without the need of passing through the minor populated state A. Therefore, the mechanism can be simply described by two possible kinetic routes: (i) the “induced fit route” (IF route), where predominantly the open form of MBP changes into H basin directly, that is, 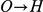; and (ii) the “population shift route” (PS route), where MBP arrives at the H basin through the A basin, that is, 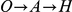. Therefore, we can infer the mechanism of conformational change of MBP based on the state-transition routes under the free energy landscape. Rigorously speaking, the PS route represents that MBP first shifts the pre-existing distribution of conformations upon ligand binding followed by local conformational adjustment.

The conformational energy landscape of MBP shows that the landscape has three major basins with one basin biased towards the open and the other two toward the apo-closed and holo-closed conformations of the protein. This indicates that the experimentally determined structures are perfectly integrated into our model. The entire conformational dynamics of the system is characterized by the inter-basin transitions, which depends on the barriers between and relative energies of basins [5]. The conformational energy landscapes are reshaped by the change of ligand concentration which can modulate the height of free energy barriers or the depth of native state basins.

**Figure 3:**
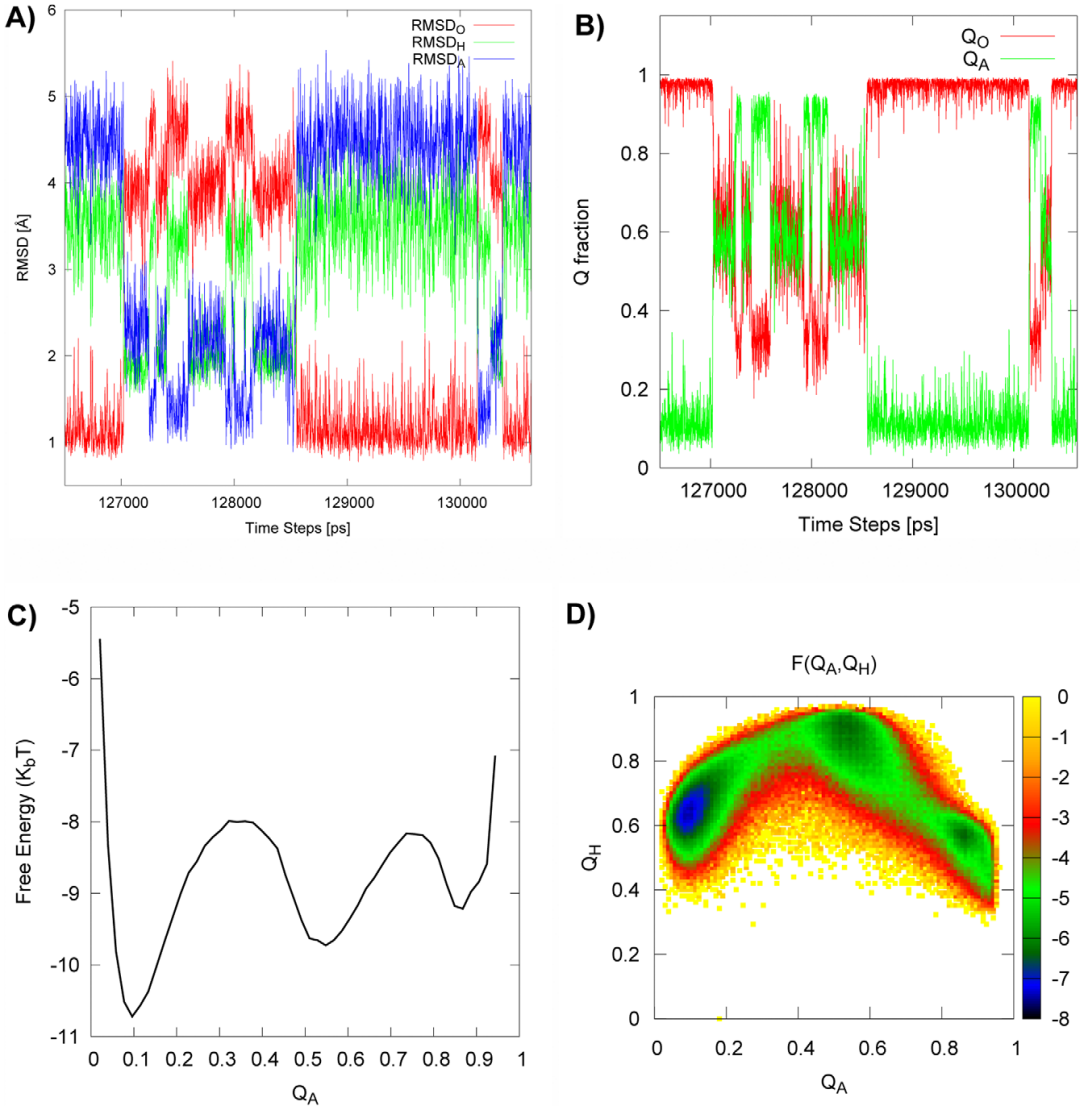
Induced fit pathway revealed by conformation switching between open, holo-closed and apo-closed states. The parameters are 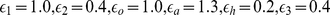, 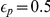, 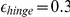, using 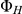 potential. (A–B) shows the typical trajectories in time for Q fractions and RMSDs. (C) the free energy profile as a function of 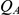. (D) the two dimensional free energy profile as a function of 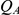 and 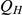. There is a direct route from the open basin to the holo-closed basin implying a clear induced fit pathway. The apo-closed basin is not on the way to the holo-closed basin, but is, in fact, off pathway. In addition, we can identify two transition states 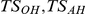 between O, A, H basins which are located at (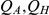) = (0.3,0.8) and (0.7,0.65).

In Figure 3A–B, the typical time trajectories as a function of RMSDs (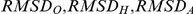) and Q fractions (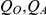) are shown. It indicates transitions between open and holo-closed states, and between holo-closed and apo-closed states. In addition, Figure 3C–D show the free energy profile as a function of 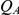 and the two-dimensional free energy profile as a function of 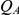 and 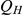. We found a direct route from open basin to holo-closed basin clearly implying an induced fit pathway. The apo-closed basin is not on the way to holo-closed basin, but is, in fact, circumvented en route.

### Hidden population shift pathways revealed by kinetic analysis

By analysing the kinetic trajectories, we found the evidence that PS routes exist which are hidden in thermodynamic free energy profiles (Figure S5 in *Text S1*). A typical kinetic PS route in MBP is shown in Figure S6 in *Text S1*. A thermodynamically invisible path may actually exist. Under certain conditions related to reactant concentrations and rate constants, the flux through a given pathway can quantitatively reflect a reaction proceeding along the respective path [45].

From the energy landscape perspectives, the induced fit is a limiting case of conformational selection when the interaction partner selectively binds to the lowest energy conformation [3]. Hence, the actual mechanism in each allosteric protein may reside between the two limits. In many cases, the allosteric pathway may be modulated by the ligand and protein concentrations, so that both mechanisms may occur at certain conditions [46]. To precisely measure how much a transition proceeds through a given route, we calculate the reactive flux through that route. From our calculation, only O
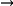
H transition flux (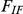) and O
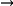
A
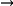
H transition flux (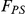) are computed to analyse the relative importance of two limiting mechanisms when a ligand binds to MBP.

Furthermore, we investigated the dependence of fractional flux of IF route (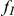) and PS route (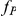) on the ligand concentration and hinge flexibility. Note that a comparison of the effects with different parameters should be performed under the same conditions. The fractional flux 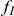 and 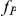 can be calculated as follows:
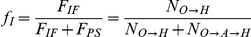


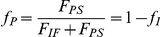
where 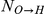 is the number of direct transition from the open basin to the holo-closed basin without passing through any other basins. 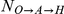 is the number of sequential transitions from the open basin to the apo-closed basin and then arriving at the holo-closed basin.

The basin dynamics of the system was investigated by applying the method analogous to the one described in [47] that explored the folding free energy landscape. We construct a reduced kinetic scheme by considering the transitions just between the native basins. The transition rate constant for this model, for example 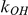, is estimated as the ratio of transitions number 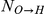 to total residence time in open basin. Generally, 
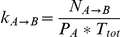
, where 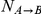 is the the number of transitions from basin A to B, 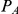 is the probability of protein in basin A, 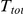 is the duration of the trajectory. Note that 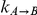 is in unit of 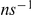.

**Figure 4:**
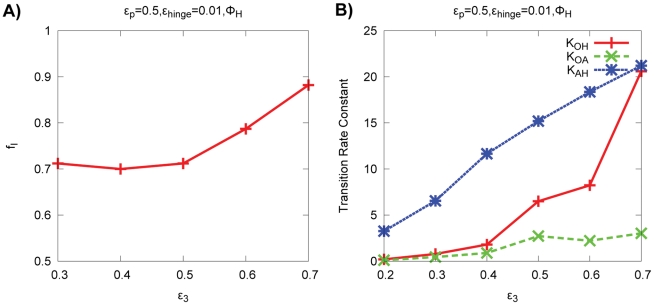
Influence of ligand concentration on kinetic mechanism and transition rate constant. (A) Fractional flux of IF route (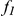) as a function of ligand concentration. Increasing ligand binding interactions can facilitate MBP to activate its conformation following an induced fit pathway. Intriguingly, 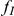 is always kept on high values (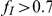) during all the ligand concentrations. This strongly supports that the induced fit pathway is the predominant activation route of the system. (B) Transition rate constants 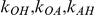 as a function of ligand concentration. 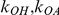 and 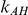 increased as ligand concentration increased. However, 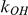 increased more sharply than 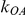 and 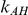. Our simulation results give strong support that if the transition rate between ligand-free major state and ligand-binding active state is sufficiently high, then the enzyme will mostly follow the direct conformational transition route, resulting in a predominant induced fit mechanism.

We next considered the kinetic response of MBP to changes in ligand concentration. The relationships of the fractional flux and transition rate constant with 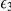 are shown in Figure 4. From Figure 4A, it is clear that the IF transition flux increases with increasing 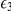, especially for slightly higher values (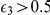). The correlation between 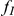 and 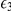 indicates that the increasing ligand binding interactions encourage MBP to follow an induced fit pathway. This finding is consistent with the conclusions by Hammes et al. [45] that increasing ligand concentration favors the induced fit pathway. Intriguingly, 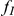 is always kept at relatively high values (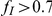) for all ligand concentrations. This strongly supports that the induced fit pathway is the predominant activation route of the system. The contribution of PS routes to the total activation flux does not exceed 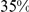 (Figure S7 in *Text S1*). As pointed out by Zhou [46], the conformational transition rates are also a key factor in controlling the population of pathways. Figure 4B indicates that the transition rate constants 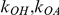 and 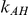 increase as ligand concentration increases. However, 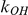 increases more sharply than 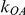 and 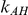. This can be easily understood considering that while the conformational transitions between the open and the holo-closed basin are fast, MBP can quickly switch to the active state directly from the open state. Our simulation results indicate that if the transition rate between the ligand-free major state and the ligand-binding active state is sufficiently high, then the protein will mostly follow the direct conformational transition route that results in a predominant induced fit mechanism.

The relationships between kinetic behaviors and ligand concentration were studied using 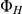 model (Figure 4). To alleviate the model dependence caused by the effects of the dihedral barrier of the hinge regions in different dihedral models, we employed parameter 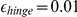. Note that 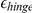 is introduced to rescale the energetic contribution of the angle term for the hinge regions. Thus, this parameter can be used to tune the hinge flexibility. The effects of the hinge flexibility on kinetics are discussed in detail in the following section.

**Figure 5:**
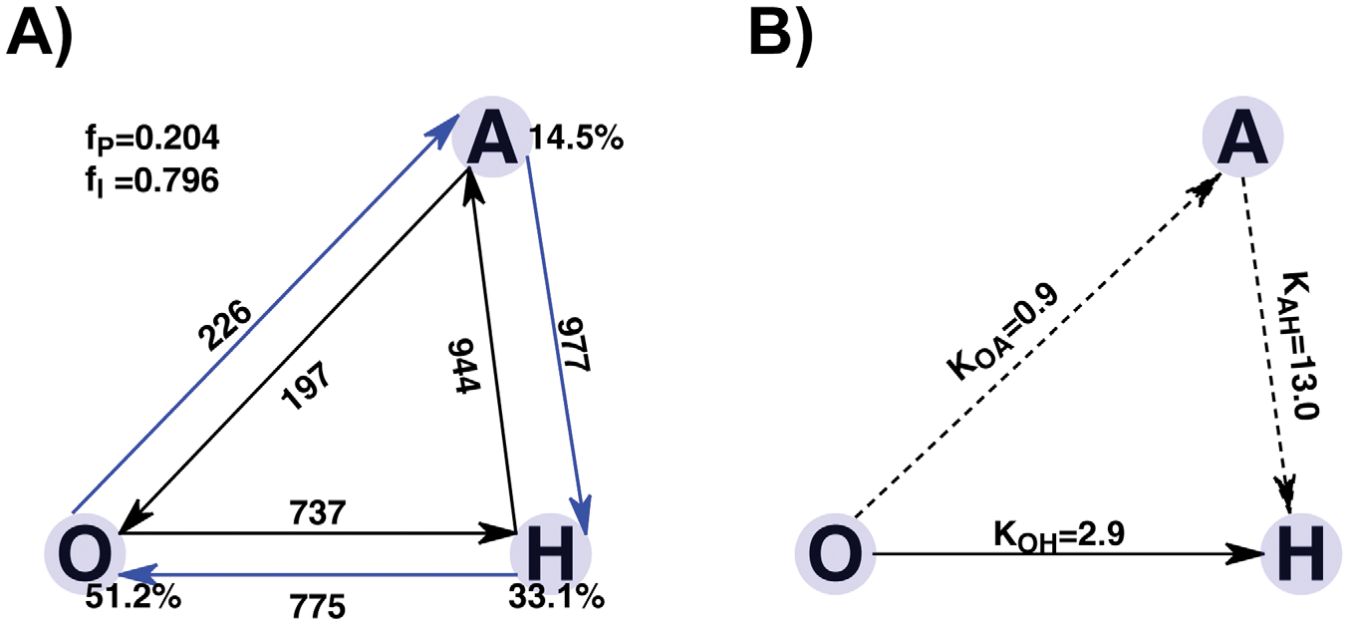
Schematic representation of the basins dynamics. 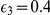
 and 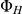 model is used. The other parameters are 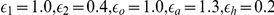, 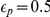, 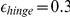. Open, apo-closed and holo-closed states are labeled by O, A and H respectively. (A) Transitions between these basins are represented by arrows along with transition numbers. Transitions that occur clockwise are represented as blue arrows. The basin probability 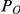, 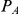 and 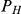 are 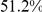, 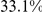 and 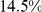 respectively. The fractional flux of the IF route is 0.796. It means that there are 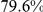 of open to holo-closed transitions that proceed through the induced fit pathway, whereas the rest of the 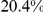 of transitions follow population shift pathway. (B) Rate constants (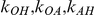 in unit of 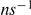) of conformational change are shown. Direct O-H transition is represented by solid arrow, O-A and A-H transitions by dashed arrows. 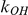, 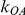 and 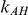 are 2.9, 0.9, 13.0, respectively. The rate of A-H transition is about 4 times the O-H transition rate, and 15 times the O-A transition rate.

In particular, we analyze the detailed kinetic trajectories generated by the specific model with 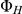 potential using a parameter set with moderate values (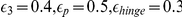). Note that the corresponding free energy profiles under the same model are shown in Figure 3. Figure 5A shows the schematic representation of the basins dynamics. The transitions between the basins are represented by arrows along with the transition numbers. Transitions that occur clock-wise are represented as blue arrows. The fractional flux of IF route is 0.796. It means that 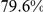 of open to holo-close transitions proceed through induced fit pathway, the rest follow population shift pathway. The basin probabilities 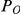, 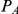 and 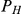 are 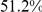, 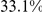 and 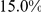 respectively. The percentages of basin probability do not necessarily add up to exactly 100% because of minor misfolding conformations outside these native basins.

Moreover, for the timescale of the transitions between the open state and the apo-closed state, the experiments suggest an upper limit of 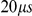 [48] and a lower limit of 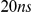 [33]. In addition, the O
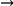
H transition rate can be estimated around 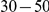 ns [36] by experimental association rate constants that fall within a very narrow range of 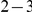
×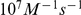 [49], despite the differences in the size and nature (linear and cyclic) of the maltodextrin ligands. In our simulations, the activation transitions from ligand-free MBP is illustrated in Figure 5B in which 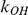, 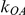 and 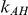 are 2.9, 0.9, 13.0 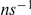, respectively. It should be noted that, in our coarse grained structure-based model, transitions between native basins are significantly faster than the realistic time scale. The absolute time scales cannot be obtained due to the coarse grained nature of our model and the lack of explicit solvent molecules. It is, however, possible to make an order of magnitude estimate based on the gap of time scale between simulations and experiments. Accordingly, it may be reasonable to scale the simulation time by two or three order of magnitude. This transformation should be taken as a very crude order of magnitude estimate.

Clearly, the rate of A
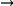
H transition is about 4 times the O
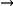
H transition rate, and 15 times the O
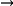
A transition rate. The finding that 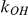 is larger than 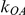 in our model is supported by the fact that the open to holo-closed transition can be observed in MD simulations that are at least 30 ns long [35], and the pre-existing equilibrium in apo MBP between an open and a partially closed conformer was only observed after 300 ns of simulation time [36]. The true transition rates should be dependent on a number of factors, such as temperature, pH, and ligand concentration. Here, they are calculated under a condition corresponding to modest ligand concentration (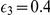). The results indicate that the relatively less contribution of PS routes is due to the fact that O
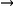
A transition is the rate-limiting step, although the protein transitions into the H basin from the A basin more quickly than from the O basin.

### Influence of hinge flexibility on thermodynamics and kinetics

It is widely accepted that enzyme activity is closely related to the fast, local fluctuations reflecting the flexibility of mostly the hinge regions. These hinges were computationally identified from differences in both pseudo-angles and pseudo-dihedral angles between native states (see details in section Model and Methods in *Text S1*). In the following, we will investigate the influence of hinge flexibility on the thermodynamics and kinetics of the system.

First, the influences of hinge flexibility on thermodynamics are summarized in Figure S8 in *Text S1*. Considering the different native angle biases of 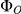 and 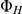 model, we compared the results to assess the model dependence. With 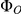 model, the hinge flexibility (the decrease of 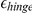) is found to mainly decrease the depth of the open basin, by destabilizing the open-closed basin and increasing the stability of the apo and holo-closed basins, but has little influence on either 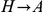 or 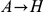 transition barrier. With 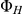 model, it shows that hinge flexibility mostly decreases the stability of holo-close basin and increases the stability of apo-closed basin. However, there is little impact on 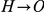 free energy barrier and stability of open basin. Although the models may have different native biases, they all imply that increasing hinge flexibility can decrease the free energy barriers from their native basin to other basins.

Furthermore, we investigate the effects of hinge flexibility on kinetics (Figure S7 in *Text S1*). From the correlations between fractional flux of IF routes and hinge flexibility, we can see that the increase of hinge rigidity in both models facilitates the conformational transition of MBP along the induced fit pathway. The PS routes are more favored for high hinge flexibility as the transition between the open and the apo-closed conformations is not only accompanied by domain rotation, but also by significant domain twist, both of which depend on hinge flexibility.

From the relationships between transition rate constants and hinge flexibility, we can see that in 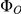 model, increase of hinge flexibility can greatly accelerate the O
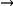
H transition. However the corresponding fractional flux 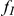 decreases as hinge flexibility increases. This can be explained as the competition between IF transitions and PS transitions as 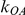 and 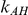 also increase. To be more exact, 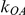 increases 17.3 times and 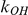 increases 10.6 times from 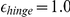 to 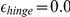. In contrast, in 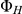 model, the O
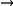
H transition process is slightly slowed down when increasing the hinge flexibility, and the corresponding fractional flux also decreases.

Overall, the flexibility of hinge regions play an important role in both basin stability and basin dynamics.

### Structural characterization of transition states

The above thermodynamics and kinetics results reveal a mixed mechanism for the conformational change in MBP upon ligand binding. It consists of a major induced fit route and a minor population shift route. We located the transition state by finding the extremum between the minima of the free energy profile as the saddle point of the free energy landscape. From the thermodynamic free energy profiles in Figure 3D, we can identify two transition states 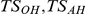 between the O, A, H basins which are located at (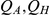) = (0.3,0.8) and (0.7,0.65). And from Figure S4 in *Text S1*, another transition state 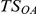 between the O and A basin can be identified and located at (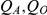) = (0.6,0.5). First, we describe the structural information in the transition state ensembles.

**Figure 6:**
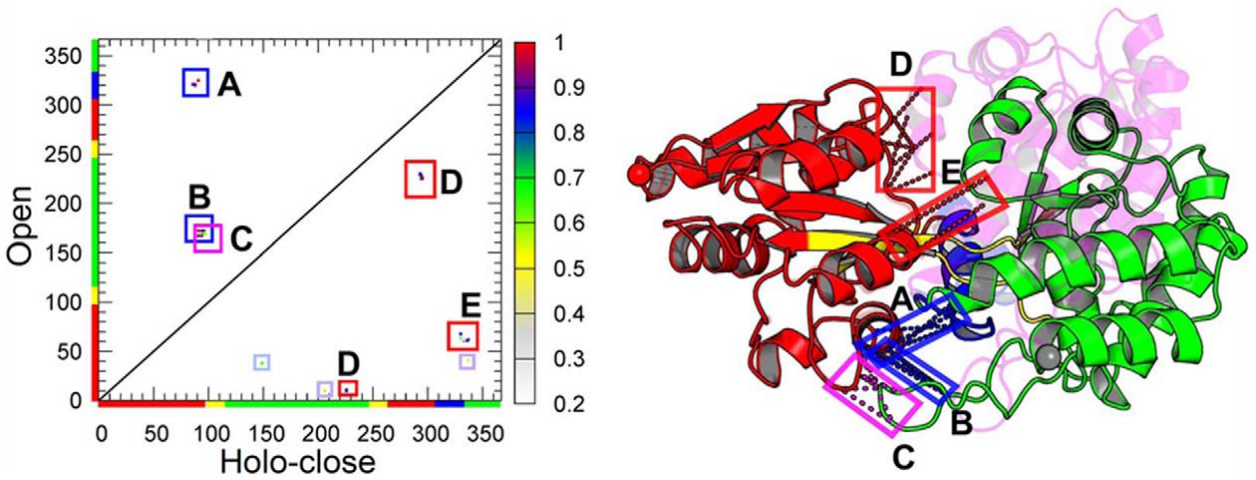
Structural characterization of transition state ensembles 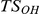
 **.** Native state-specific contacts with different probability formed in 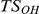 are mapped onto the open and holo-closed crystal structures. In the left column, it shows the residues' contact probability maps. Note that, we only show the state-specific contacts because most of contacts are shared by the two-ending transition basins. The upper triangle (lower triangle) corresponds to probability map of the O-specific (H-specific) native contacts. The corresponding probability for a particular residues' pair forming two-body native contact is illustrated according to the side color bar in which dark colors mean high formation probability and light colors mean low formation probability. The segments corresponding to the NTD (1–94 and 259–310, red), CTD (113–250 and 331–366, green), linker1 (102–112 and 250–260, yellow) and linker2 (310–330, blue) are labelled in the X-axis (holo-closed state) and Y-axis (open state) with different colors corresponding to the structures of the right column. In the right column, the state-specific contacts are mapped onto the three-dimensional structures of MBP. Note that the NTD is used to superimpose open and closed conformations, and the CTD for holo-closed structure is represented by transparent cartoon in red. Red squares (D and E regions) and magenta squares (C regions) highlight the H-specific interactions formed and the O-specific interactions broken in 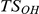 respectively. Blue squares (A and B regions) label the O-specific interactions that are not broken. The H-specific contacts not formed in TS are labeled by grey squares. They are not mapped onto the structures for clarity.

**Figure 7:**
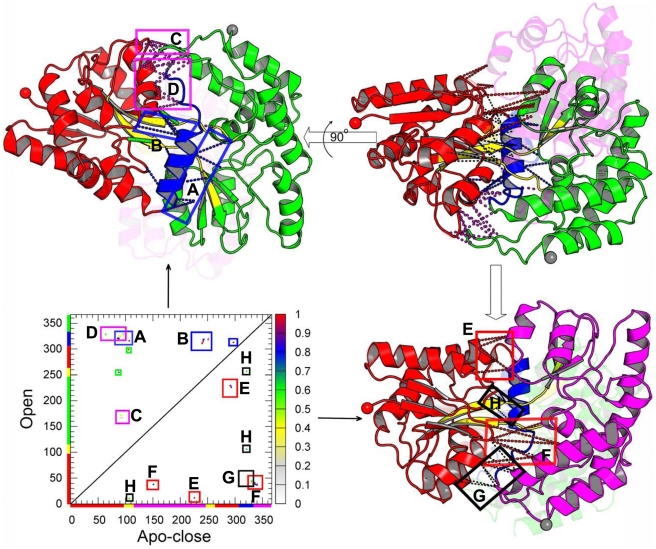
Structural characterization of transition state ensembles 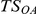
 **.** Native state-specific contacts with different probability formed in 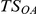 are mapped onto the open and apo-closed structures (upper right subfigure). For the contact probability map (lower left subfigure), the upper triangle and lower triangle correspond to probability map of the O-specific and A-specific native contacts, respectively. Note that the NTD is used to superimpose the open and closed conformations. For clarity, the O-specific mapped structures and A-specific mapped structures are shown separately in upper left and lower right corners respectively. For O-specific mapped structures (upper left subfigure), which are turned 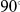 for the illustration of the back of active site groove, the CTD for apo-closed structure is represented by transparent cartoon in red. For the A-specific mapped structures (lower right subfigure), the CTD for the open structure is represented by the transparent cartoon in green. Blue squares (A and B regions) label the O-specific interactions which are not broken in 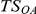. These broken O-specific interactions are highlighted by magenta squares (C and D regions). Red squares (E and F regions) and black squares (G and H regions) highlight the formed and unformed A-specific interactions respectively. The green squares in the contact probability map and green dashed lines in the mapped structure are used to label the O-specific contacts (F88-V255 and V106-A299) between linker1 and NTD at the bottom of the ligand bind cleft, which are not broken in TS.

In Figure 6 and 7, we show the contact probability maps for transition state ensembles of 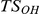 and 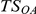. We map these contacts onto the three-dimensional structures of MBP. Note that only the state-specific contacts are illustrated. This is due to the fact that most of the contacts are shared by the two-ending transition basins. Their probabilities are shown by points with different colors according to side color bar in which dark colors mean high probability and light colors mean low probability.

Figure 6 shows the structural characterization of the transition state between the open and holo-closed states. Clearly, we can see that the contacts between the tip of balancing loop and NTD (C region) are broken and the interface between NTD and CTD at the tip of ligand binding cleft (D region) forms 7 native H-specific contacts (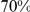 formation). Note that H-specific contacts represent the contacts that are specificial for the holo-closed state. A-specific denotes contacts for the apo-closed state and O-specific denotes contacts for the open state. By contrast, the interface at the tail of cleft (E region) only forms 2 contacts (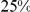 formation), and the long-range H-specific contacts whose optimal distances in open state are larger than 20 Å are not formed. However, the contacts between balancing the linker and NTD (A region) and part of contacts between balancing the loop and NTD (B region) are not broken. We propose that the balancing loop movement switches the closure of the tip of ligand binding cleft and the balancing linker motion triggers the final closure of the cleft in open to holo-closed transition.

Figure 7, shows the structural characterization of 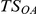. We can see that contacts (C region) between the balancing loop and NTD are broken and a number of contacts (D region) between the balancing linker (mostly located at residues K322 to I329) and NTD are also broken. The interface at the tip of cleft (E region) forms all A-specific native contacts (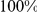 formation). A part of A-specific contacts at tail interface (F region) is formed and the rest (G region) is not formed. In addition, the O-specific contacts between helix part of balancing linker and two domains (region A and B) are not broken. Comparing 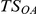 with 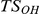, we found that more contacts between the balancing interface (containing the balancing loop and balancing linker) and NTD are broken in 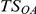, and more contacts are formed between the interface at the ligand binding cleft. It seems that not only the balancing loop acts as a switch that initiates the domain closure, but also the balancing linker plays an important role in the conformational change whose role was not investigated in the earlier simulation [36].

Note that A-specific contacts in H-region contain pairs D10-E107, K11-E107, V106-Q321, V257-Q321 which represent the interactions between linker1 and NTD and between linker1 and linker2 in apo-closed state. These contacts are not formed in TS. The green squares in contact probability map and green dashed lines in mapped structure are used to label the O-specific contacts (F88-V255 and V106-A299) between linker1 and NTD at the bottom of the ligand bind cleft, which are not broken in TS. It indicates that the domain twist does not occur in 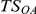 because that the twist has to disrupt the O-specific contacts F88-V255 and V106-A299 and may form A-specific contacts (in H region) between linker1 and the loop1 (N8-K11) in NTD (such as, ion pair K11-E107). From this point, we propose that domain partial closure occurs before domain twist in the transition between the open and apo-closed states.

### Functional 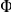 values and local cracking in transition states analysis

Characterization of the transition state properties, especially the inhomogeneous distribution of contacts between residues, will help us to understand the microscopic structural mechanism of conformational change through locating the sites or seeds for the nucleation. The 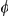 values provide important characterization for particular residues at the transition state ensemble. By experimental 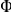 value analysis, one can identify the critical residues with high values that cluster together in the transition state to form the nucleus for conformational change from the unfolded state to the native folded state. In our model, we use functional 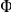 values to characterize the transition state ensemble for conformational changes between multiple basins [6]. See details of functional 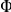 values calculation in *Text S1*.

We calculate the functional 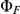-values 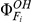 and 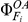 for particular residues with a sensible difference in the thermal mean number of interaction contacts in the transition states of 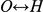 transition and 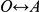 transition. The results are summarized in Figure S9 in *Text S1*.

It shows that certain residues in NTD (D10, K11, K293, P294, L295) and in CTD (P225, W226, S229) play a key role in stabilizing the holo-closed state (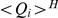 is larger than 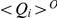) and contribute to the closure of MBP (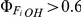). These residues are clustered together in the holo-closed state to form a tightly interacting network corresponding to the high contact probability region in Figure 7. Our model also predicts that the 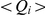 values of residues F88, D91, R94, N96, Y167, N169, Y172 and Q321 in 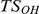 are especially close to their 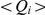 in open state (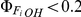). This can also be explained by the contact probability map where O-specific contacts (A region) between balancing linker and NTD and partial O-specific contacts (B region) between balancing loop and NTD are intact. In addition, the 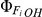 values for residues Y95, N96, G170, K171 (white small spheres) are about 0.5 and 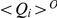 values for them are larger than 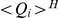, indicating that the interactions between the balancing loop and NTD are partially broken in 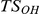. This is consistent with the result reported from contact probability maps.

The 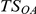 analysis indicates that the clustered residues are the same as in 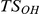 except that K38 also plays an important role in stabilizing the apo-closed state. In addition, the 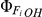 value for E107 is 0.7, which indicates that E107 also plays a favorable role in stabilizing the apo-closed conformation in 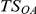. These interactions may include the salt bridge between K11 and E107 as a member of the “hook-and-eye” motif, which can be important to lock the protein-ligand complex in a semiclosed conformation [35]. E107 is located at the base of the binding cleft, in the middle of linker1. Compared to the open state, the side-chain carboxylate 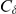 shifts 3.4 Å in ligand-bound closed state and 6.2 Å in apo-closed state thus moving up into the cleft. Furthermore, for helix A15 in C2 domain (see secondary structural definition in *Text S1*), there are not only high 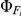 residues (W336, Y337, R340), but also low a 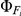 residue (M332). This is consist with the contact probability map in Figure 7 that shows part of native contacts in the apo-closed state is formed and part of them is absent in the interface at the tail of the ligand binding cleft. The high 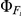 values for residues in the balancing loop (N169 and G170) indicates that the interactions between NTD and the balancing loop are completely broken as 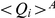 are close to 0 for F88, D91, N96, G97, N169 and G170.

Interestingly, we found that 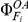 values of K252, K322 and G323 are larger than 1 (yellow spheres) as 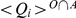 is out of the range of 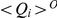 and 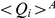. For K252, 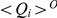, 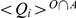 and 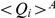 are 3.8, 2.8, 3.3 respectively. For K322, they are 3.9, 2.6, 3.1, and 2.7, 1.6, 2.2 for G323. K252 is located at the loop region of linker1, and K322 and G323 are located at the loop region of linker2 (balancing linker). Their abnormal 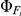 values indicate that the movements of linker1 and linker2 are not completely coupled during the open to apo-closed transition. Especially, for the loop region in linker2, the large structural fluctuation may cause it to move away from linker1, resulting in loss of contacts between K252 and K322 and G323. On the other hand, the loss of contacts in linkers brings about more flexibility for rigid-body domain movement. Such behaviour of breaking and reforming native contacts is known as “backtracking”. It is somewhat surprising that such a “backtracking” phenomenon is observed during the exchanging process of predominantly open form and minor partially closed form in MBP. To our knowledge, it is the first reported case in protein allostery, although such behavior has been been observed in protein folding [50]–[52] and binding [41]. Such functional “backtracking” behavior is in favor of conformational change.

It is well known that large structural arrangement of protein can be realized by changes of several dihedral angles at the hinge regions. In some cases the high strain energy may accumulate in various localized regions. It can be relaxed by local unfolding or cracking if the energy exceeds a threshold [7]. See the detailed calculation of local unfolding in *Text S1*. The results of local cracking calculation are summarized in Figure S10 in *Text S1*. Our model indicates that the local unfolding points contain V106, L117, P119, E149, E168 and N328 in 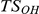. And for 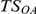, these unfolding regions include H60, I104, P150, Q148, T204, P244, K322, G323, M326. These residues are located at the loop regions with high flexibility with the exception of I104 and V106 approaching to the known hinge residue E107. They are located at linker1. The high 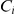 value (see definition in section of Local Cracking in *Text S1*) for E168 which is located at the balancing loop may be caused by the high flexibility of balancing loop that are not in contact with NTD. In addition, N328 in 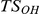 and K322, G323, M326 in 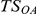 are located at the loop region of the balancing linker.

In MBP, the hydrophibic residues have been found to be important to the domain closure [30], [32], [36], which makes the protein more compact by reducing the solvent accessibility. When the balancing interface loses contacts with NTD, the side-chain of non-polar residues become more solvent-exposed, which in turn drives a distant conformational change in active site cleft. More specifically, our simulation reveals that the solvent exposure of the balancing loop drives the tip of active site cleft to close. We also found that the charged residues are important, however, compared to 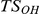, the electrostatic interactions seem to be more important for 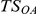 in which four charged residues (D37, K38, E41, R340) take part in the contact networks formed at the tail of active site cleft. Among these contacts, there is a salt bridge D37-R340 formed between NTD and CTD. Another inter-domain salt bridge K38-E149 can be found specifically in the holo-closed state, but it is not formed at 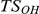. In addition, the functional 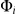 value analysis reveals that E107 may help stabilizing the 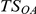 through the formation of salt bridge between K11 and E107. Taken together, we propose that the population shift pathway is more electrostatically driven than the induced fit pathway for MBP.

### Dependence of MBP unfolding on ligand binding

We further explored the relationship between unfolding characteristics of MBP and ligand concentration through simulation. The thermodynamics of unfolding in MBP has been previously explored by a combination of differential scanning and titration calorimetry and fluorescence spectroscopy under different solvent conditions [53]. In this work, we simulated the folding dynamics using our functional model. The results are summarized in Figure S11 in *Text S1*. It indicates that the specific heat curves shift to higher temperatures with increasing ligand concentration, suggesting an increase of protein stability. In addition, the folding cooperativity increases as well, which is illustrated by the narrowing of the specific heat profile.

Structurally, MBP contains two globular domains. Between the two domains, it forms a deep groove whose base locates the ligand-binding site. The X-ray structure of MBP in complex with maltotriose [24], [26], [54] shows that the binding pocket is lined with a number of polar and aromatic groups from both domains that participate in hydrogen-bonding and van der Waals interactions with the ligand. These interactions make the two interlaced domains more tightly packed against each other. Overall, our simulations support that the presence of a ligand increases the cooperativity and stability of MBP because the ligand binding bridges the two domains more tightly. Higher ligand concentration or binding with ligands with higher affinity, makes the protein not only more stable, but more cooperative. This conclusion is in agreement with earlier experimental findings [53].

## Discussion

Conformational transitions are central to a multitude of physiological processes, such as enzyme catalysis, and also essential for nonenzymatic binding events. Due to the limit of current experimental techniques, the molecular details for the microscopic structural mechanism of conformational change are often lacking. Therefore we developed a theoretical model for describing the structure-function relationship, and extended the double-well model [5]–[10], [12]–[17], into a triple-well model, which we used to account for conformational switching between the open, apo-closed and holo-closed states of MBP.

It is worthwhile noting that the microscopic mixed double-well models with two reference conformations may construct additional basins in the free energy landscape, such as in Lu and Wang's microscopic double-well model [5] and in Whitford's mixed contact map model [6]. However, these models don't necessarily yield the required free energy basins. For example, Lu and Wang's microscopic double-well model can generate four free energy basins for ADK [5], but no additional basins beside the two reference basins for glutamine-binding protein (GlnBP) [55]. In fact, we have attempted to build a dual-basin model, which only integrated the open and the holo-closed conformations of MBP. However, this model failed to generate the apo-closed basin, demonstrating the limitations of these double-well models in simulating complex multi-state systems. Such limitations are what led us to develop the present triple-basin model.

Constant temperature molecular dynamics simulations were performed to obtain the free energy landscape and kinetics for conformational changes. It clearly reveals an induced fit pathway on the free energy surface. In addition, a minor population shift pathway is found through the analysis of kinetic trajectories. By introducing the flux analysis as suggested by Hammes et al. [45], we measured the relative weight of the two parallel activation paths in a quantitative way. The results support a mixed mechanism of MBP in the presence of moderate concentration of ligand whereby major induced fit pathways and minor population shift pathways coexist.

The thermodynamic and kinetic manifestations of function transitions are closely related due to the correlation between transition rates and free energy barriers of basin hopping. Rigorously speaking, the population shift route (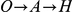) represents the population shift followed by local conformational adjustment (induced fit) similar to some other systems [16], [56], [57]. The mixed mechanism is consistent with the free energy landscape sampled by atomic simulation [37], [38], where the ligand binding induced conformational change is proposed to be dominant for MBP. In our work, we quantified the relative weights of both induced fit and population shift from kinetic pathway perspectives. These results support the view that the underlying mechanism of conformational transition does not necessarily follow the population shift scenario, and that even free protein populates a wide range of conformations.

In nature, conformational change of protein upon ligand binding can be explained by either one of the two mechanisms or their combinations. It is dependent on the systems under different conditions. The relative weights of both pathways are dependent on a number of factors, including the ligand and protein concentrations, the conformational transition rates, and the properties of ligands etc. [11], [45], [46], [58]. Other methods also have been developed, such as Markov state models (MSM) [57]. Our work provides a good example of quantifying the relative contributions of multiple possible mechanisms.

A “Venus Fly-trap” model was employed to explain the conformational change of PBPs family [59]. The hinge regions are believed to play a key role in control of the rapid response of PBPs upon ligand binding resembling that the carnivorous plant traps its prey [60]. In the present work, we investigate the role of the hinge in conformational change, especially for MBP. We found the flexibility of hinge regions plays an important role not only in the basin stability and but also in basin dynamics. The results also support that increasing the flexibility of the hinge regions promotes the population shift route.

It is still a matter of debate whether it is a general rule that dynamical equilibrium between the open and the minor closed conformation exists for all the free PBPs though our current and past work certainly supports this assertion [33], [37], [38], [57], [61], although Bermejo et al. claimed that there was no such apo-closed state for GlnBP [61]. The findings in our simulation support the explanation that the population shift mechanism is unfavourable for conformational change of GlnBP due to the fact that its hinge relatively rigidified by strong hydrogen bond interactions.

Protein folding is critical for three dimensional structural formation, and the conformational switching between multiple well defined states is vital for interactions and functions at the molecular level. They are often studied separately. So far, there have been abundant studies to explore the structure-folding relationship [9], [62]. Recently, more and more attention has been turning to the functional transitions and native dynamics of proteins [5], [6], [17], [34], [38]. However, there are very few studies to investigate the interplay between protein structure, folding, and function as a whole. In this work, we explored the folding landscape and functional landscape of MBP under the same theoretical framework using our triple-well structure-based model. To our knowledge, this work is the first study to provide such a description. In addition, the effects of ligand concentration in protein melting were also investigated. The conclusion that the presence of ligand increasing the cooperativity and stability of MBP agrees well with earlier experimental findings. The success of reproducing experimental folding properties also implies that the coarse-grained implicit ligand model has the ability to capture the essential effects of ligand binding not only on functionally conformational change but also on the global folding landscape.

**Figure 8:**
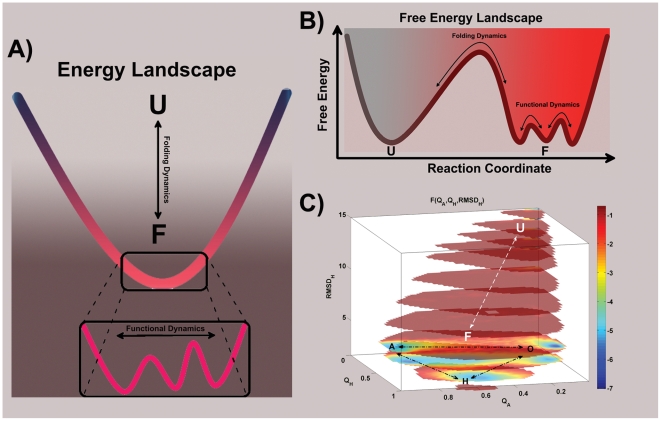
Schematic and actual representations of the folding landscape and functional landscape. A schematic energy landscape and a schematic free energy landscape are shown in (A) and (B), respectively. The unfolded state and folded states are denoted as U and F, respectively. (C) The actual free energy landscapes is shown by the high-dimensional free energy profile as a function of 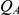, 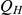, and 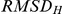 (in unit of Å) under modest ligand concentrations (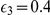). The dashed arrows represent functional transitions between the open, apo-closed and holo-closed states. The 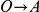 route that is hidden in two-dimensional free energy profiles can be visible in the four-dimensional free energy profiles. For better view of the functional landscape, the conformational regions with 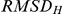 beyond 15 Å are not shown.

From the perspective of energy landscape theory, protein folding and protein function share a common funneled energy landscape [63] (Figure 8A). Proteins fold their conformation into low energy ensembles from the top of the funnel to the bottom, and carry out the biological function by changing their conformations between a modest number of folded states in response to ligand binding or environmental changes. Compared with the whole folding phase space, the number of conformations is much smaller in the functional landscape. It makes the bottom of the funnel more easily sampled using current computational resources, especially with the aid of coarse grained models. We can consider protein folding as the large-scale (inter-basin between native and non-native) conformational changes between unfolding state and folded states, and functional transitions as small-scale (intra-basin, native) conformational changes between multiple folded basins. Although the folding dynamics were usually sampled by structure-based models with a single native basin, in principle, these multi-basin models were specially developed for functional dynamics should have the ability to capture the folding dynamics, as done in the present work.

Furthermore, the physical reason why unfolding/melting occurs in the functional study can be answered using the the thermal energy scale (Figure 8B). When the temperature is high (in fact melting temperature is much higher than the functional transition temperature), the energy scale 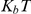 is high. At this scale, the differences between global energy landscape of double well and triple well for function and single well funnel for folding are blurry and even indistinguishable. Conformational dynamics at this temperature in these models all look like large-scale folding/unfolding transitions between the top of the energy funnel and the bottom (native basins). At the low temperatures, corresponding to the low energy scale near native states, the conformational dynamics occur at the bottom of landscape (functional landscape), the difference between folding funnel model and functional multi-basin model will be explicit and distinct. In other words, on the coarsed grained level, we have a funneled energy landscape for folding. If we dive into the smaller scales of the energy landscape near the native states, we see the more detailed structure and functional energy landscape emerging (Figure 8C).

There have been several theoretical studies about conformational dynamics of MBP, most recently [36]–[38], [64] stimulated by the experimental finding of hidden partially closed conformation [33]. There are substantial differences between these studies and the present work. Our model is based on energy landscape theory in which proteins minimize the conflicting interactions so as to be minimally frustrated and have globally funneled energy landscapes biasing towards their native structures as a consequence of evolution [34]. It is well established that the folding mechanism and binding mechanism are encoded in the topology of proteins in the native folded state [9]. Therefore, our model is developed based on the assumption that the functional transitions are mostly governed by protein topology. Our model is structure based, which can give both statistically reliable thermodynamic and kinetic information at the coarse grained level, while others that are more detailed atomic based giving statistically sampling limited thermodynamic information but no kinetic information.

Although, electrostatic interactions are taken into account in our model, it may be true that other energetic frustrations also contributing to the roughness of energy landscape. There are several limitations in our models. The water solvent molecules are not explicitly considered here. Furthermore, the ligand is also implicitly introduced by ligand-mediated contacts. The implicit modeling of the ligands has an advantage in simplification of simulation and interpretation of the results, however it is unable to precisely account for the local nature of the interactions between ligand and protein. For example, the beta-cyclodextrin does not elicit domain closure for MBP, except for interactions with only the CTD [65]. In our simulation, we introduced the ligand-mediated interactions between NTD and CTD to reflect the ligand binding. So, our simulation is only able to represent the binding effect for these physiological ligands (such as maltose, maltotriose) with the exception of beta-cyclodextrin which interacts with CTD only. This important feature is also missing in the other models [5]–[10], [12]–[15]. Indeed, we have further refined this model by introduction of explicit ligands. The improved model has been applied in this protein and another allosteric system and the related works will be submitted to be published soon.

Taken together, we propose that the conformational dynamics of MBP can be unraveled by assuming a triple-basin energy landscape (whose depths are modulated by ligand) that corresponds to distinct but related topological states. This work demonstrates that a multiple native basin biased landscape which follows the principle of minimal frustration is sufficient to fold and function with complex topologies. From this perspective, we extend the application of the funneled energy landscape that explains how most proteins fold efficiently and robustly to their structures in functional transitions. Furthermore, we expect that our model can be extended to address more complex allosteric systems.

## Materials and Methods

Depending on how pairwise interactions between residues are treated, current multiple-basin structure-based models can be broadly divided into two categories: microscopic and macroscopic. In macroscopic models, techniques borrowed from the Marcus theory of electron transfer [66], [67] or an exponential Boltzmann-weighting method [12] are used to construct a double-well potential with two smoothly linked energy basins. In these models, pairwise favorable interactions in each reference structure are biased to its respective native basin. In contrast, with microscopic models [4], [5], [13], the two reference energy surfaces are integrated into two-well potentials for each pair of specific interactions. This results in a rougher landscape with more possible macroscopic states other than native basins. In addition, there is another method [6] that belongs to the type of models between microscopic and macroscopic level. In this approach, a potential based on one reference structure (ligand-free state) was modified by the addition of a bias favoring interactions present in another reference structure (ligand-bound state). The energy potential in the model is constructed by combining multiple single-well potentials without redundant interactions. In other words, it mixes the contact maps rather than modifying the form of potential function. In the present work, we developed a coarse-grained model as an extension of such a model.

### Triple-well model

To account for the side-chain dynamics, especially for active site, we developed a mixed coarse-grained model in which part of amino acids are represented by two beads. The coarse-graining process is similar to SMOG tool [68]. Based on the fact that the interface between the NTD and CTD is rich in charged residues, we introduced the electrostatic interactions into the model. In addition, we introduced a pseudo-ligand into the bound simulation by adding selected ligand-mediated interactions to the potential, an approach that has been used before in the double-well coarse-grained simulation [6], [11], [40]. The total Hamiltonian for the MBP system is given by the expression:
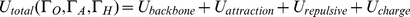
The total energy is divided into backbone, non-bonded and electrostatic interactions. The backbone interaction 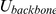 maintains the geometry and local bias. The non-bonded interaction can be partitioned into two components, an attraction term 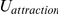 to provide the triple-basin bias by a mixed contact map and a repulsive term 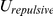 to provide the excluded volume.

For the last term 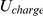, we used the Debye-Huckel potential to introduce the electrostatic interactions [69]. Water and ions were incorporated implicitly into the interaction model as the dielectric constant and the Debye screening length. We have tested the DH model using an extensive set of dielectric constants ranging between 40 and 160 and ion concentrations ranging from 0 to 0.20 M. The results of DH model with different parameters are summaried in Figure S12 in *Text S1*. Changing the dielectric constant or the salt concentration did not significantly affect the functional dynamics of MBP. Finally, we employed a salt concentration 0.10 M and a dielectric constant 80. All the analysis in manuscript was based on this values except where specified. Of course, the DH model is valid for low salt concentration and mainly for dilute solution. See *Text S1* for the detailed descriptions of these terms and the accompanying parameters of the force field.

### Simulation protocols

Simulations were performed with Gromacs 4.0.5 [70]. Reduced units were used for all calculations. A time step of 0.0005 time units (or ps) was used and the simulation was coupled to a temperature bath via Langevin dynamics with a coupling time of 1.0. For each individual trajectory, the total simulation time was 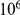 time units. To ensure that the simulation is converged and the statistical errors are small enough, we simultaneously ran several independent simulations using the same parameters set. Simulations were performed at T = 0.5, and this temperature is used throughout the article, except where specified. Systems were initialized arbitrarily in one of the three native states.

### Reaction coordinates

Q (and also RMSD) has been suggested to be useful when studying the folding landscape. Unfortunately, for functional dynamics which occurs at the bottom of the energy funnel, the traditional folding Q is unable to monitor the process due to the fact that most of the contacts shared by the native states are not needed to be broken. Fortunately, the reaction coordinates from protein folding can be borrowed to describe the functional landscape with minor modification. That is, to measure the formation of state-specific contacts instead of all native contacts. We can denote this quantity as the state-specific Q fraction or functional Q. The state-specific Q fraction can distinguish the conformational transitions between the native basins. This reaction coordinate has also been successfully used in other allosteric studies [18], [40]. See more details in *Text S1*.

## Supporting Information

Text S1 **Supporting information of multi-state functional dynamics of MBP.**
(PDF)
