# Supplementary material for: Exploration of Multi-State Conformational Dynamics and Underlying Global Functional Landscape of Maltose Binding Protein
Source: PLoS Comput Biol. 2012 Apr 19;8(4):e1002471. doi: 10.1371/journal.pcbi.1002471 (PMC3330084; doi:10.1371/journal.pcbi.1002471)
Supplement: Text S1 — Supporting information of multi-state functional dynamics of MBP. (PDF) [file pcbi.1002471.s001.pdf]

SI Text

Yong Wang [*], **Chun Tang** [†], **Erkwang Wang** [*‡], **and Jin Wang** [*§¶]

[*]State Key Laboratory of Electroanalytical Chemistry, Changchun Institute of Applied Chemistry, Chinese Academy of Sciences, Changchun, Jilin, 130022, P.R. China,[†]State Key Laboratory of Magnetic Resonance and Atomic and Molecular Physics, Wuhan Institute of Physics and Mathematics, Chinese Academy of Sciences, Wuhan, Hubei 430071, China, and [§]Department of Chemistry and Physics, State University of New York at Stony Brook, Stony Brook, New York 11794-3400, USA

Model and Methods

Energy Hamiltonian. To extend a structure-based model to systems with multiple basins, we modified the contact map and the dihedral angles. Note that all the residue indexes are rearranged from 1 to 366 in the model. To include the side chain dynamics on the conformational change of enzyme, we developed a coarse-grained model in which each amino acid is represented by one or two beads dependent of its location. One bead (named C_A in our model), representing the backbone, is located on the C_α atom, and one bead (name C_B), representing the side chain, is located on the center of mass or the farthest heavy atom of the side chain, depending on its residue characterize (see details in section of *Side-chain Interactions*). Based on the fact that the interface between the NTD and CTD is lined by negatively charged residues, that are responsible for an array of hydrogen bonds with the sugar substrate in holo-close form of MBP, we introduced the electrostatic interactions into the two-bead model to study the effect of charged residues on the conformational change of MBP. The two-bead structure-based hamiltonian is given by the expression:

$$
\begin{aligned}
&U_{total}(\Gamma_O, \Gamma_A, \Gamma_H) \\
&\quad = U_{backbone} + U_{attraction} + U_{repulsive} + U_{charge} \\
&\quad = \sum_{bonds} K_r (r - r_0)^2 \\
&\quad + \epsilon_p * [U_{angle}(\gamma^{non-hinge}) + \epsilon_{hinge} * U_{angle}(\gamma^{hinge})] \\
&\quad + \epsilon_1 * U_{LJ}(\gamma^C) \\
&\quad + \epsilon_2 * [\epsilon_o * U_{LJ}(\gamma^O) + \epsilon_a * U_{LJ}(\gamma^A) + \epsilon_h * U_{LJ}(\gamma^H)] \\
&\quad + \epsilon_3 * U_{LJ}(\gamma^{ligand}) \\
&\quad + \sum_{non-native} K_{repulsion}(\frac{\sigma_{NC}}{r_{ij}})^{12} \\
&\quad + K_{coulomb} * B(k) \sum_{i,j} \frac{q_i * q_j * exp(-k r_{ij})}{\epsilon_r r_{ij}}
\end{aligned}
$$

The total energy is divided into backbone, non-bonded and electrostatic interactions. The backbone interaction $U_{backbone}$ maintains the geometry and local bias. The non-bonded interaction can be partitioned into two components: attraction term $U_{attraction}$ is contained within a contact map to provide the tertiary bias; and repulsive term $U_{repulsive}$ is used to provide the excluded volume. The contacts are determined from the given PDB structures. Any atoms not interacting through a contact, bond, angle or dihedral, are considered 'non-contacts' and interact only through excluded volume. The key to construct a triple-basin model is to define a mixed contact map which integrates multiple structural information together. The U_{charge} term introduces the electrostatic interactions. A detailed description of a structure-based model can be found elsewhere (1).

In the MD simulation, we used coordinates of PDB 1OMP, 2V93 and 3MBP to represent the open, apo-close and holo-close state, respectively. A triple-well potential was modelled according to the three PDB coordinates. Note that, we use the first MODEL of 2V93 to import the information of the apo-close state.

Repulsive interaction.

$$U_{repulsive} = \sum_{non-native} K_{repulsion}(\frac{\sigma_{NC}}{r_{ij}})^{12}$$

σ_{NC} is the excluded distance between non-native pairs to provide excluded volume repulsion. The repulsive radii is 4.0 and 2.5 $\mathring{A}$ for C_A and C_B, respectively. r_{ij} is the distance between bead i and bead j. The strength of the repulsive term $K_{repulsion}$ is 1 kJ/mol. All possible contact pairs existing in contact map of each state are not considered in the repulsive term.

Construction of triple-basin potential.

$$
\begin{aligned}
U_{attraction} &= \epsilon_1 * U_{LJ}(\gamma^C) + \epsilon_2 * [\epsilon_o * U_{LJ}(\gamma^O) + \\
&\quad \epsilon_a * U_{LJ}(\gamma^A) + \epsilon_h * U_{LJ}(\gamma^H)] + \epsilon_3 * U_{LJ}(\gamma^{ligand}) \\
U_{LJ}(\gamma^X) &= \sum_{\gamma^X} [5(\frac{\sigma_{ij}}{r_{ij}})^{12} - 6(\frac{\sigma_{ij}}{r_{ij}})^{10}]
\end{aligned}
$$

The non-bonded interactions are defined by contact map obtained with CSU (2). CSU acknowledges a contact between two atoms based on the intersection of their putative surfaces and their hydrophobicity. Each contact is regarded as an attractive interactions between the two involved amino acids. In our two-bead model, these interactions are divided into different contributions. The possible combinations are contacts of the type $C_A - C_A$, $C_A - C_B$ and $C_B - C_B$. In our model, we only include $C_A - C_A$ and $C_B - C_B$ contacts, although $C_A - C_B$ pairs still are included in repulsive term given by $\sigma_{ij} = \sqrt{4.0 * 2.5} = 3.16\mathring{A}$. Including the $C_A - C_B$ contacts into the model should not qualitatively change the folding mechanism (3).

The concrete details of building the mixed contact map are as follow:

a) **Total contact library γ^{All}.** We build a contact library by collecting all the $C_A - C_A$ and $C_B - C_B$ pairs in the contact maps obtained from open, apo-close and holo-close PDB coordinates. There are 1359 contacts (1313 $C_A - C_A$ pairs and 46 $C_B - C_B$ pairs) in the total library γ^{All}.

b) **Reference distances.** We calculate the distances r^O_{ij}, r^A_{ij} and r^H_{ij} between the two beads i and j in the library. r^O_{ij}, r^A_{ij} and r^H_{ij} represent the beads' distance in open, apo-close and holo-close states, respectively. The often encountered problem is these contacts appear in the three states with different

[‡]E-mail:ekwang@ciac.jl.cn

[¶]To whom correspondence should be addressed. E-mail: jin.wang.1@stonybrook.edu

r_{ij}. Thus, care must be taken while constructing a mixed attractive potential with multiple basins.

c) **Core sublibrary γ^C.** We define $R_{ij}(X/Y) = \frac{r_{ij}^X}{r_{ij}^Y}$, X and Y can be O, A and H. For a bead pair i and j, if any $R_{ij}(X/Y) < R_{cut}$, this bead pair is considered as a member of core contact library (γ^C). We set $R_{cut} = 1.1$ for $C_A - C_A$ pairs and $R_{cut} = 1.25$ for $C_B - C_B$ pairs. There are 980 $C_A - C_A$ pairs and 14 $C_B - C_B$ pairs in core contact library γ^C.

d) **State-specific sublibraries γ^O, γ^A and γ^H.** If any possible $R_{ij}(X/Y) > 1.5$ is in a pair, this pair will be considered as a member of unique contact library which contains three subsets, open library γ^O, apo-close library γ^A and holo-close library γ^H. Note that any contact libraries contain three parameters, atom indexes of two beads and corresponding optimal distance. Any other conflicting contacts which may strain the system are disregarded. There are 31 contacts (all is $C_A - C_A$ pairs) in γ^O. There are 55 contacts (47 $C_A - C_A$ pairs and 8 $C_B - C_B$ pairs) in γ^H. There are 40 contacts (23 $C_A - C_A$ pairs and 17 $C_B - C_B$ pairs) in γ^A.

In summary, γ^O, γ^A and γ^H combine the structural information of open, apo-close and holo-close state, furthermore the additional contact library γ^{ligand} are added to represent ligand binding. The strengths of the core contacts and the state-specific contacts are rescaled by ϵ_1 and ϵ_2, respectively. And the strength of ligand binding contacts is rescaled by ϵ_3. Furthermore, ϵ_o, ϵ_a and ϵ_h are introduced to modulate the relatively stability of O, A and H basins, respectively.

Side-chain Interactions. A comparison between the apo-close (PDB code 2v93) and holo-close (PDB code 3mbp) forms of MBP suggests that the side chains in active site groove play important roles in binding with maltose ligands. A C_α-backbone model with uniform interactions is lack of resolution to describe this feature. Thus, a number of important features associated with the conformational transition of MBP are involved with side-chain dynamics. To introduce side chains into a coarse-grained structural based model, we have modified the C_α model. Because many side-chain interactions in active site of MBP are involved between charges residues (Asp, Glu, Lys, Arg), we use the most distant heavy atom (N,C,O) from the C_α to model the charged residues. For the non-charged residues, the interaction centers of side chains are modeled by using center of mass of the side-chain atoms in the native structures. We did not include the side chains, except those around the active site, so that in the model to minimize the computational cost. Including other side chains into the model should not qualitatively change the dynamics.

Ligand-mediated Contacts (γ^{ligand}). We introduce a pseudo-ligand into the bound simulation by adding selected ligand-mediated interactions to the structured based potential, an approach that has been used before in the double-well coarse-grained simulation (4, 5). An explicit coarse-grained ligand representation was previously used for simulation of ligand-induced global transitions in the catalytic domain of protein kinase A (6). However, such a representation will make the dynamics more complex.
i) We select the residues around ligand in the active site.
It includes D10, K11, E40, W58, E149, P150, Y151, W226, M326, W336, Y337, R340 (Fig. S1).
ii) We calculate the distance r_{ij} between the C_α atoms of any two ligand-binding residues i and j (in the above list).
iii) We exclude local contacts ($|i - j| < 4$ residues) and intra-domain contacts.
vi) The ligand-mediated contacts library γ^{ligand} is constructed by the contacts in the holo-close coordinate with C_A-C_A distances that are over 40% further apart ($R_{ij}(O/H)$ or $R_{ij}(A/H) > 1.4$) while in the open form or apo-close form. Finally, we identified 14 ligand-mediated interactions based on the maltotriose-binding close structure (3MBP).

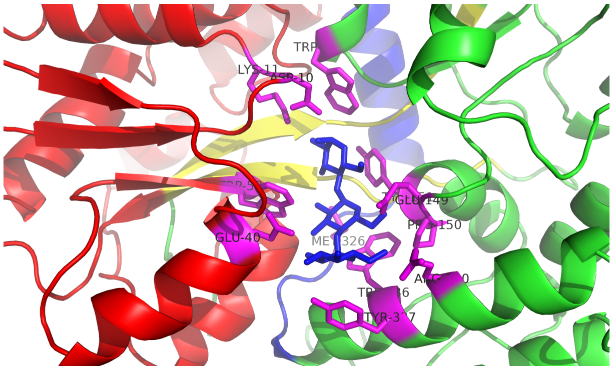

Fig. 1. Important residues around active site. Ligand (maltotriose here) is represented by blue sticks.

Backbone Potential and the Flexibility of Hinge Regions.

$$
\begin{aligned}
U_{backbone} &= U_{bond} + \epsilon_p * [U_{angle}(\gamma^{non-hinge}) + \\
&\quad \epsilon_{hinge} * U_{angle}(\gamma^{hinge})] \\
U_{bond} &= \sum_{bonds} K_r (r - r_0)^2 \\
U_{angle}(\Gamma_X) &= U_{CX-CX-CX}(\Gamma_X) + \\
&\quad U_{CX-CX-CX-CX}(\Gamma_X) \\
U_{CX-CX-CX}(\Gamma_X) &= \sum_{angles} K_\theta (\theta - \theta_0)^2 \\
U_{CX-CX-CX-CX}(\Gamma_X) &= \sum_{dihedrals} K_\phi [(1 - cos(\phi - \phi_0)) + \\
&\quad 0.5(1 - cos(3(\phi - \phi_0)))]
\end{aligned}
$$

The total backbone energy is divided into bond stretching, angle bending, torsion interactions. The bonded energies U_{bond} are summed over the energy of all co-valent bonds. $K_r = 10000$ kJ/(mol nm^2) is the bond constant, r is the distance between the two bonded atoms and r_0 is the reference distance of these atoms in the native structure. The angular energy U_{angle} has the angle constant $K_\theta = 20$ kJ/mol. θ is the angle between two adjacent bonds, and θ_0 is the reference angle in the native structure.

The dihedral energy $U_{dihedral}$ possesses the dihedral constant K_ϕ and the angle ϕ between the two planes formed by four connected atoms. We distinguish between different dihedral angles, dependent on the type of the involved atoms. If all four atoms are C_A, K_ϕ=0.344 kJ/mol. For $CB-CA-CA-CB$ dihedral angles and $CA-CA-CA-CB$ dihedral angles, K_ϕ=0.086 kJ/mol. Improper torsion angles (or improper dihedral angles) are implemented using the same equation. Improper dihedral angles have a maximum value at the cis conformation and help to maintain the system's chi-

rality.

The hinge regions were computationally identified from differences in both pseudo-angles and pseudo-dihedral angles between any two native conformations of open, apo-close and holo-close states. Hinges are located wherever pseudo angle or pseudo dihedral angle differences are greater than threshold values. In this article, the threshold values for angle bending term $U_{CX-CX-CX}(hinge)$ is 15 degree and for dihedral angle term $U_{CX-CX-CX-CX}(hinge)$ is 40 degree corresponding to $1KJ/mol$. The list of hinge regions is shown in Fig. S2. Note that we only list the backbone angles (consecutive CA-CA-CA) and dihedral angles (consecutive CA-CA-CA-CA) in table.

Pseudo-angles Table in Hinge Regions

i-1	i	i+1	O	A	H	|O-A|	|A-H|	|O-H|
250	251	252	109.11	120.02	103.6	10.91	16.42	5.51
253	254	255	111.89	132.06	101.58	20.17	30.48	10.31
323	324	325	119.08	113.24	130.78	5.84	17.54	11.7
327	328	329	95.376	111.52	94.328	16.144	17.192	1.048

Pseudo-dihedral angle Table in Hinge Regions

i-1	i	i+1	i+2	O	A	H	|O-A|	|A-H|	|O-H|
252	253	254	255	256.96	292.03	245.48	35.07	46.55	11.48
309	310	311	312	210.39	176.14	217.57	34.25	41.43	7.18
322	323	324	325	264.17	55.12	250.82	150.95	164.3	13.35
327	328	329	330	109.68	93.39	134.05	16.29	40.66	24.37

Fig. 2. Tables of hinge regions determined according to our criterion. Only the backbone angles (consecutive CA-CA-CA) and dihedral angles (consecutive CA-CA-CA-CA) are listed in table.

Electrostatic Interaction.

$$U_{charge} = K_{coulomb} * B(k) \sum_{i,j} \frac{q_i * q_j * exp(-kr_{ij})}{\epsilon_r r_{ij}} \qquad [1]$$

The electrostatic potential was represented by the Debye-Huckel model, which is a linearization of the Poisson-Boltzmann equation. All charged residues were given a full charge according to their electrostatic charge at neutral pH. The charge was placed on the C_B bead. The Debye-Huckel theory predicts the range of electrostatic influences of an ion to be the Debye screening length $1/k$. Linearization of the Poisson-Boltzmann equation yields the following relation $k^2 = \frac{8\pi N_A e^2 \rho_A}{1000\epsilon k_B T}$, where N_A is Avogadro's number, ρ_A is the solvent density, e is the proton charge, ϵ is the solvent dielectric constant, and C_s is the ionic concentration in molar unit. For monovalent salt at room temperature and with $\epsilon = 80$, $k = 0.32\sqrt{C_s}\mathring{A}^{-1}$. q_i is the point charge of the ith bead, r_{ij} is the distance between two charged beads, $K_{coulomb} = 4\pi\epsilon_0 = 332kcalmol^{-1} = 138kJmol^{-1}nm^{-1}$.

B_k is the salt-dependent coefficient, and r_{ij} is the distance between charged beads i and j. For the calculation of the screening factor k and the salt-dependent coefficient B(k), solvent density was taken as 1kg/L and the ion radius was taken as 1.4 $\mathring{A}$. More details of the Debye-Huckel model can be found elsewhere (7, 8).

In this study, we set $\epsilon_r = 80$, the dielectric constant of water is at near-ambient conditions. The Debye screening length $1/k$ depends on the salt concentration. For simplicity, we focus on physiological salt concentrations of 100 nM, which lead to $1/k = 10\mathring{A}$. Residue charges correspond to pH 7, such that $q_i = +e$ for Lys and Arg, $-e$ for Asp and Glu, where e is the elementary charge.

Superior Angle Models. Based on the difference of backbone angles in all possible states of MBP, we developed three models: Φ_O (using native angles in open state), Φ_A (using native angles in apo-close state) and Φ_H (using native angles in holo-close state). We found that Φ_O and Φ_H models were able to reproduce the three native states with reasonable stability. It seems that the energy landscape constructed from Φ_A potential is not smooth enough to make the system mis-fold in local minima other than native basins. This result suggests that the apo-close structure as a relatively unstable conformation, is not appropriate to be used as the basic structure to model the basin dynamics.

Functional Φ values

The functional Φ values can be approximately calculated through the following equation:

$$\Phi_{F_i}{}^{RP} = \frac{< Q_i >^{R\cap P} - < Q_i >^{R}}{< Q_i >^{P} - < Q_i >^{R}}$$

where $< Q_i >$ is the thermal mean value of the number of two-body interaction contacts for residue i over all the corresponding states, and the $R \cap P$ subscript represents the transition state from R to P state. R and P subscripts represent reactant state and product state which can be any two of the open, apo-close and holo-close states, respectively. Note that, for these residues in which the difference between $< Q_i >^P$ and $< Q_i >^R$ is less than a certain cutoff value (using 1.0 in this article), their functional Φ_{F_i} values are not calculated.

It is important to clarify the similarities and differences in the definition and interpretation of Φ_F-values and protein folding Φ-values. Protein folding Φ-values give a measure of the interaction strength of a particular residue existing in native state which is also present in the folding transition state ensemble. Similarly, functional Φ-values measure the interaction strength involving a particular residue in the transition state from R state to P state. Note that, the reactant and product states are dependent on the definition. Both folding Φ and functional Φ values give a quantitative detection of a residue's importance in the transition state from reactant to product conformation. The main difference is, in the definition of reactant state and product state, that folding Φ calculation uses the unfolded state as reactant state and folded native state as product state. Overall, $\Phi_i = 1$ implies that this particular residue is crucial for change from reactant to product state, or is close to the product conformation at the transition state. On the other hand, if $\Phi_i = 0$, then it means this particular residue is not important for change from reactant to product state or close to the reactant state at the transition state. It is noteworthy that the best targets for mutations to perturb the transition rates are those residues for which Φ_{F_i} close to either 0 or 1.

Due to the complexity of conformational switching between multiple basins, $< Q_i >^P$ can be less than $< Q_i >^R$ which indicates that contacts formed in residue i at R state are more than P state. And $< Q_i >^{R\cap P}$ can be out of the range of $< Q_i >^P$ and $< Q_i >^R$, although this case does not occur frequently. It can cause Φ_{F_i} value to be negative or even larger than 1 which are not easily interpreted in traditional folding Φ value analysis. Therefore, the functional Φ_F-values should be handled carefully.

Local Cracking

In this article, local unfolding was measured by calculating the average deviation in backbone dihedral angle comprised of residue i-1, i, i+1, i+2:

$$C_i^{RP} = 0.5 * \frac{|<\Psi_i>^{R\cap P} - \Psi_i^R| + |<\Psi_i>^{R\cap P} - \Psi_i^P| - |\Psi_i^R - \Psi_i^P|}{|\Psi_i^R - \Psi_i^P|}$$

where Ψ_i is the mean value of the dihedral angle for residue i over all the corresponding state, and the $R \cap P$ subscript represents the transition state between R state and P state. It is noteworthy that the C_i^{RP} we calculated reflects the potential cracking regions which may not contain the known hinge residues, such as E107, V257 and A308, due to the large $|\Psi_i^R - \Psi_i^P|$. Furthermore, we don't consider the residues whose $|\Psi_i^R - \Psi_i^P|$ are less than a certain threshold value (20 degree in the article). If C_i^{RP} is larger than 0.1, a local cracking is considered to occur.

Reaction Coordinates

The state of MBP is quantitatively measured by the fraction of formation of certain state-specific contacts, the so-called state-specific Q fraction or functional Q. The state-specific Q fraction has been used in previous conformational change studies (4, 9) and suggested to be a good reaction coordinate to capture the process of conformational change between multiple states. Namely, to monitor the closeness to apo-close state, we used a set of residue pairs that are in contact in the A state, but not in contact in the O state or the H state. These A-specific contacts monitor the closeness to the A state.

$$Q = \sum_{Open, Apo, Close} \frac{1 - (R_{ij} - D_0)/R_{ij}^{nat})^n}{1 - ((R_{ij} - D_0)/R_{ij}^{nat})^m}$$

Where, m=20, n=10, R_{ij}^{nat} is native distance of a pair atom i and atom j. $D_0 = 0.42 * R_{ij}^{nat}$.

Static Structure Analysis

The previous studies (10, 11) on the conformational equilibria between open and holo-close states show that interactions on the interface opposite the ligand binding cleft, located at some distance from the ligand binding pocket, are responsible for modulating the stability of the open conformation. It has an effect on the ligand-binding affinity by a "conformational coupling" mechanism (10). This functional interface, called "balancing interface", was considered to play a role of "molecular switch" that triggers the conformational turnover.

The balancing interface includes two important segments. One is a loop region (residues Y167 to D173) which is modestly conserved in MBPs from various organisms. While MBP is in open state, this loop interacts with NTD (N-terminal domain) to form a number of contacts. These contacts are broken in partially and fully closed state, resulting in higher flexibility for the loop (12, 13). Another segment is the linker2 (residues D310 to P330) which plays important roles not only in bridging the two domains, but also as the base of the ligand-binding groove. Several site-specific chemical modifications in this region, such as replacement of I325 with larger bulky groups (10), mutations of residues M317 and Q321 to alanine (11), have large impacts in destabilizing the open conformation and increasing the ligand binding affinity by almost two orders of magnitude. As such, in the present work, we term the loop as "balancing loop" and the linker2 as "balancing linker" for more clear description of the process in details (see schematic diagram in Figure 1 in main text).

By comparing the structures of open, apo-close and holo-close states, we found that balancing loop forms contacts with NTD in open state and is free in apo-close and holo-close states (Figure 1 in main text). On the other hand, from open state to holo-close state, balancing linker moves away from NTD (5.6Å translation for C_α in Q321). This results in the contacts between balancing linker and NTD decrease, and several residues (including I325, M317, Q321) become more solvent-exposed. In the apo-close state, balancing linker is closer to NTD following a rotation of linker1 and linker2. However, the interacting site in NTD is different with that in open state due to the movement of linker2.

References

1. Clementi, C, Nymeyer, H, & Onuchic, J. N. (2000) Topological and energetic factors: what determines the structural details of the transition state ensemble and "en-route" intermediates for protein folding? an investigation for small globular proteins *J Mol Biol* **298**, 937–953.

2. Sobolev, V, Sorokine, A, Prilusky, J, Abola, E. E, & Edelman, M. (1999) Automated analysis of interatomic contacts in proteins *Bioinformatics* **15**, 327–32.

3. Oliveira, L. C, Schug, A, & Onuchic, J. N. (2008) Geometrical features of the protein folding mechanism are a robust property of the energy landscape: a detailed investigation of several reduced models *J Phys Chem B* **112**, 6131–6.

4. Daily, M. D, Phillips, G. N, & Cui, Q. A. (2010) Many local motions cooperate to produce the adenylate kinase conformational transition *J Mol Biol* **400**, 618–631.

5. Whitford, P. C, Miyashita, O, Levy, Y, & Onuchic, J. N. (2007) Conformational transitions of adenylate kinase: Switching by cracking *J Mol Biol* **366**, 1661–1671.

6. Hyeon, C, Jennings, P. A, Adams, J. A, & Onuchic, J. N. (2009) Ligand-induced global transitions in the catalytic domain of protein kinase a *Proc Natl Acad Sci USA* **106**, 3023–3028.

7. Azia, A & Levy, Y. (2009) Nonnative electrostatic interactions can modulate protein folding: Molecular dynamics with a grain of salt *J Mol Biol* **393**, 527–542.

8. Givaty, O & Levy, Y. (2009) Protein sliding along dna: dynamics and structural characterization *J Mol Biol* **385**, 1087–97.

9. Yao, X. Q, Kenzaki, H, Murakami, S, & Takada, S. (2010) Drug export and allosteric coupling in a multidrug transporter revealed by molecular simulations *Nat Commun* **1**, 117.

10. Marvin, J. S & Hellinga, H. W. (2001) Manipulation of ligand binding affinity by exploitation of conformational coupling *Nat Struct Biol* **8**, 795–798.

11. Telmer, P. G & Shilton, B. H. (2003) Insights into the conformational equilibria of maltose-binding protein by analysis of high affinity mutants *J Biol Chem* **278**, 34555–34567.

12. Quiocho, F. A, Spurlino, J. C, & Rodseth, L. E. (1997) Extensive features of tight oligosaccharide binding revealed in high-resolution structures of the maltodextrin transport chemosensory receptor *Structure* **5**, 997–1015.

13. Tang, C, Schwieters, C. D, & Clore, G. M. (2007) Open-to-closed transition in apo maltose-binding protein observed by paramagnetic nmr *Nature* **449**, 1078–U12.

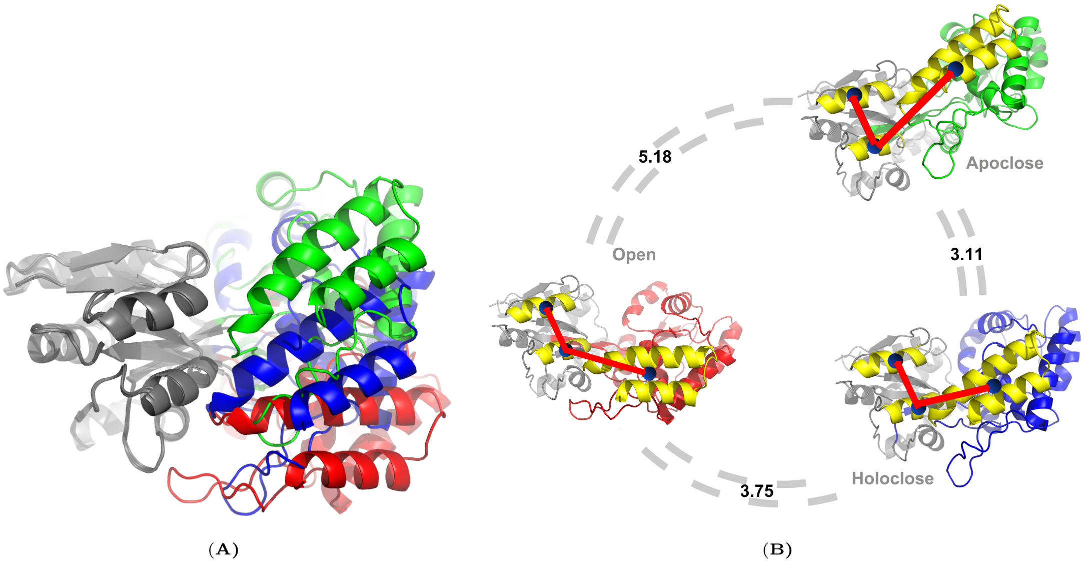

Fig. 3. Structure comparison (A) Structure comparison between open, apo-close and holo-close whose CTDs are colored in red, green and blue, respectively (NTD is grey). (B) Backbone RMSD between open and holo-close, between open and apo-close, and between apo-close and holo-close are 3.75, 5.18 and 3.11, respectively.

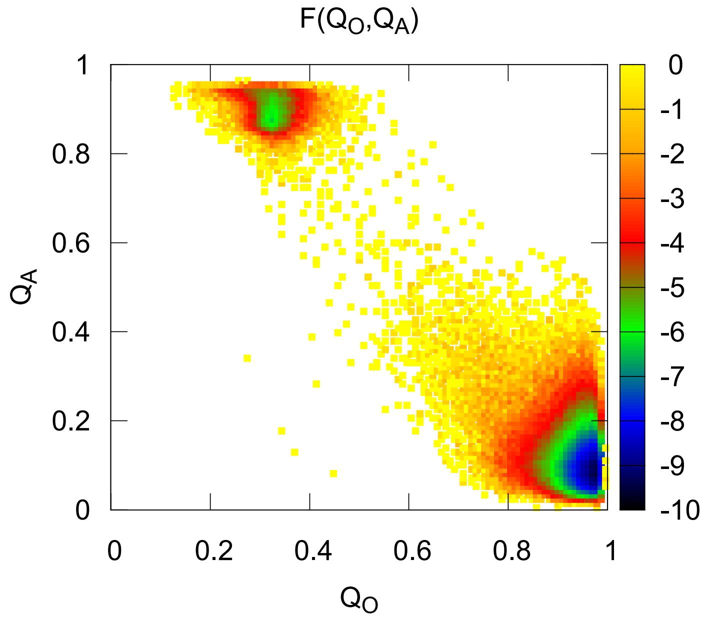

(A)

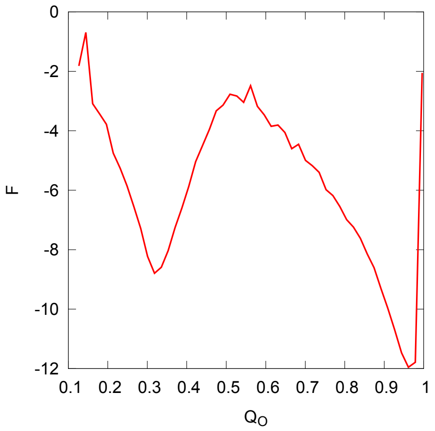

(B)

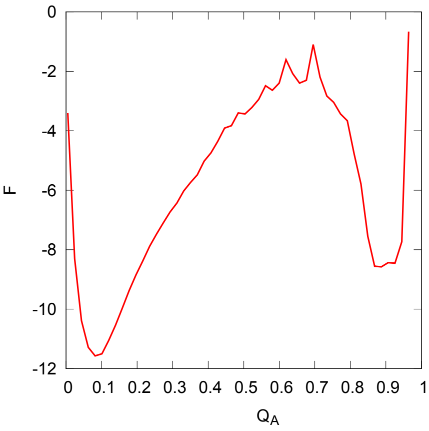

(C)

Fig. 4. Dynamical equilibrium between major open form and minor apo-close form of free MBP. These parameters are $\epsilon_1 = 1.0, \epsilon_2 = 0.4, \epsilon_o = 1.0, \epsilon_a = 1.0, \epsilon_h = 0.2, \epsilon_3 = 0.001$, $\epsilon_p = 0.5$, $\epsilon_{hinge} = 0.1$, with Φ_H potential. The population of open state is 93.96%. Apo-close state is 4.93%. Note that there is about 1.00% misfolded conformation. (A) The two-dimensional free energy surface is shown as a function of Q_O and Q_A. (B) The free energy profile is shown as a function of Q_O. (C) The free energy profile is shown as a function of Q_A. (Q_A, Q_O)=(0.9,0.3) and (0.1, 0.9) for the apo-close minimum and open minimum, respectively. The free energy barrier from apo-close to open state is $6K_bT$, from open state to apo-close is $9K_bT$. In addition, transition state TS_{OA} between O and A basin is located at (Q_A, Q_O)=(0.6,0.5).

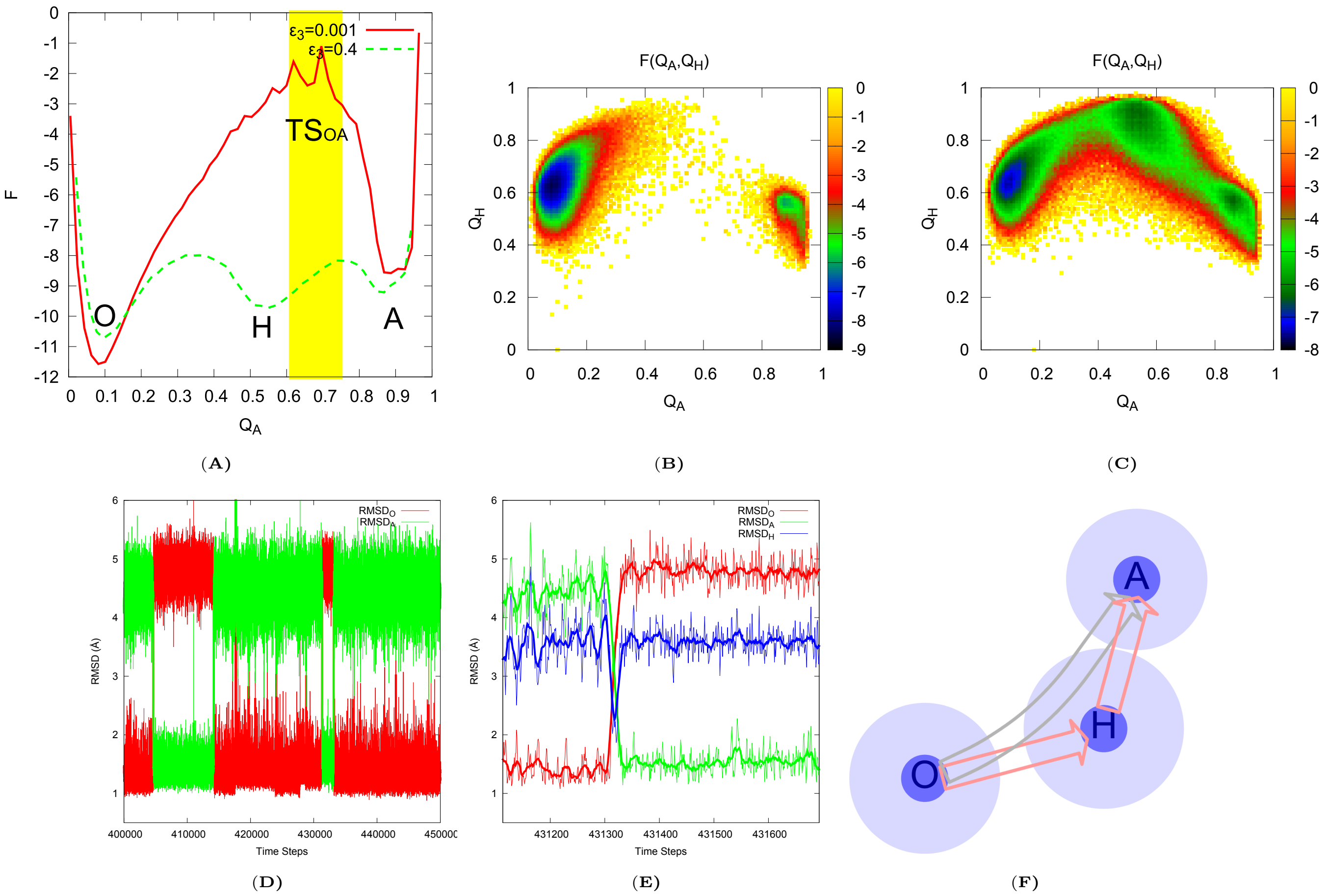

Fig. 5. (A) Free energy as a function of Q_A without ligand binding ($\epsilon_3 = 0.001$ by red solid line) and with modest ligand binding ($\epsilon_3 = 0.4$ by dashed green line). The region of transition state ensemble of O-A transition are colored in yellow. It is evident that transition state of O-A transition is not holo-close state. (B) Free energy profiles $F(Q_A, Q_H)$ of free MBP indicates O-A transition. (C) Free energy profiles $F(Q_A, Q_H)$ of MBP under modest ligand concentration indicates O-H and H-A transition. O-A transitions are hidden in the free energy profile. (D) Typical kinetic trajectory of O-A transitions. (E) One typical O-A transition. The smoother line is a running average of neighbor 20 samples. (F) Schematic basin transitions. It is difficult to distinguish the directly O-A transition (grey arrow) and indirectly O-A transition (pink arrows) passing H basin from free energy profiles.

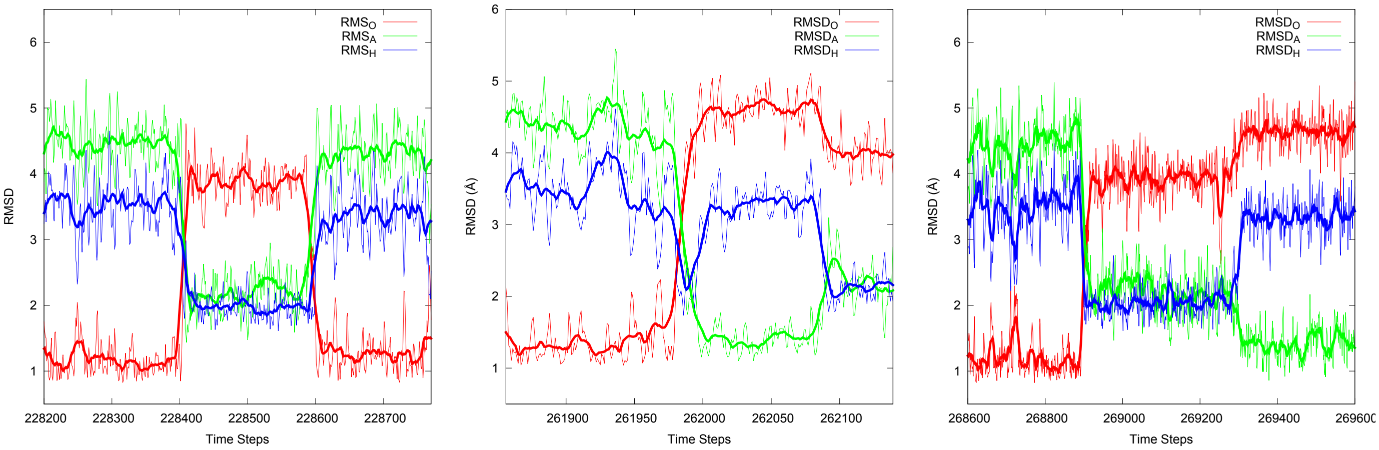

(**A**) (**B**) (**C**)

Fig. 6. Typical kinetic trajectories. (A) Induce fit route $O \rightarrow H$. (B) Population shift route $O \rightarrow A \rightarrow H$. The kinetic trajectory shows that a thermodynamically invisible path actually exist. (C) $O \rightarrow H \rightarrow A$ route.

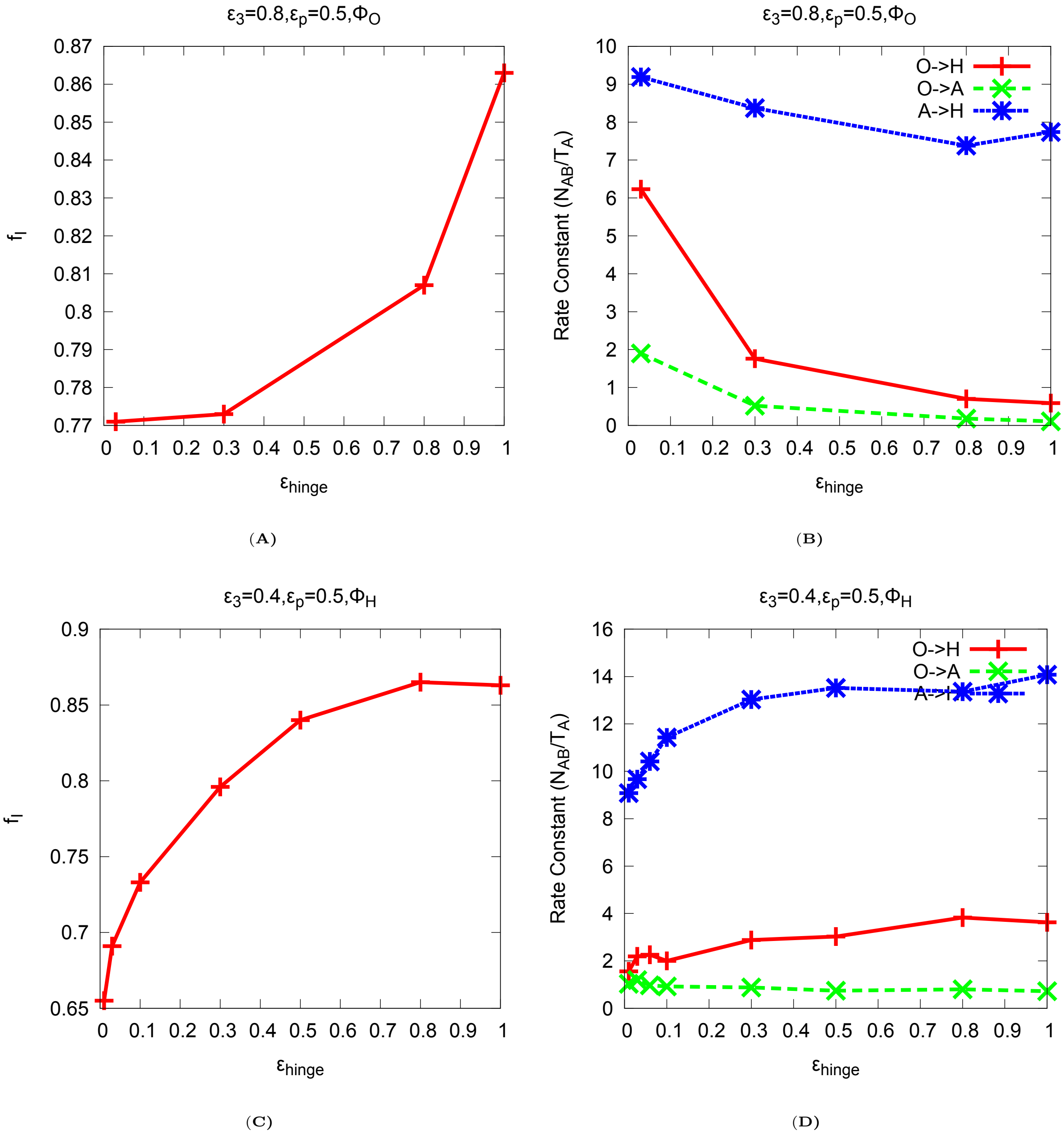

Fig. 7. Effects of hinge flexibility on the kinetics mechanism and transition rate constants. The relationships between fractional flux of IF routes f_I and hinge flexibility with Φ_O (a) and Φ_H models (c) are shown. Furthermore, the influences of hinge flexibility on transition rate constants are shown in (b) with Φ_O model and (c) with Φ_H model. Increasing hinge rigidity in both models can facilitate the conformational transition process of MBP along induced fit pathway. However, the influences of hinge flexibility on the transition rate have big differences. In Φ_O model hinge flexibility can greatly accelerate the O-H. However the corresponding fractional flux f_I decreases as hinge flexibility increases. This can be explained as the competition between IF transitions and PS transitions because K_{OA} and K_{AH} also increase. To be more exact, K_{OA} increases 17.3 times and K_{OH} increases 10.6 times from $\epsilon_{hinge} = 1.0$ to $\epsilon_{hinge} = 0.0$. In contrast, in Φ_H model, the O-H transition process is slightly slowed down by the increase of the hinge flexibility, the corresponding fractional flux decreases also. The decrease of O-H transition and the slightly increase of the rate of O-A transition which is the rate-limiting step of PS routes together lead to the decrease of fractional flux of IF routes. Overall, the flexibility of hinge regions play an important role not only in the basin stability and but also in basin dynamics.

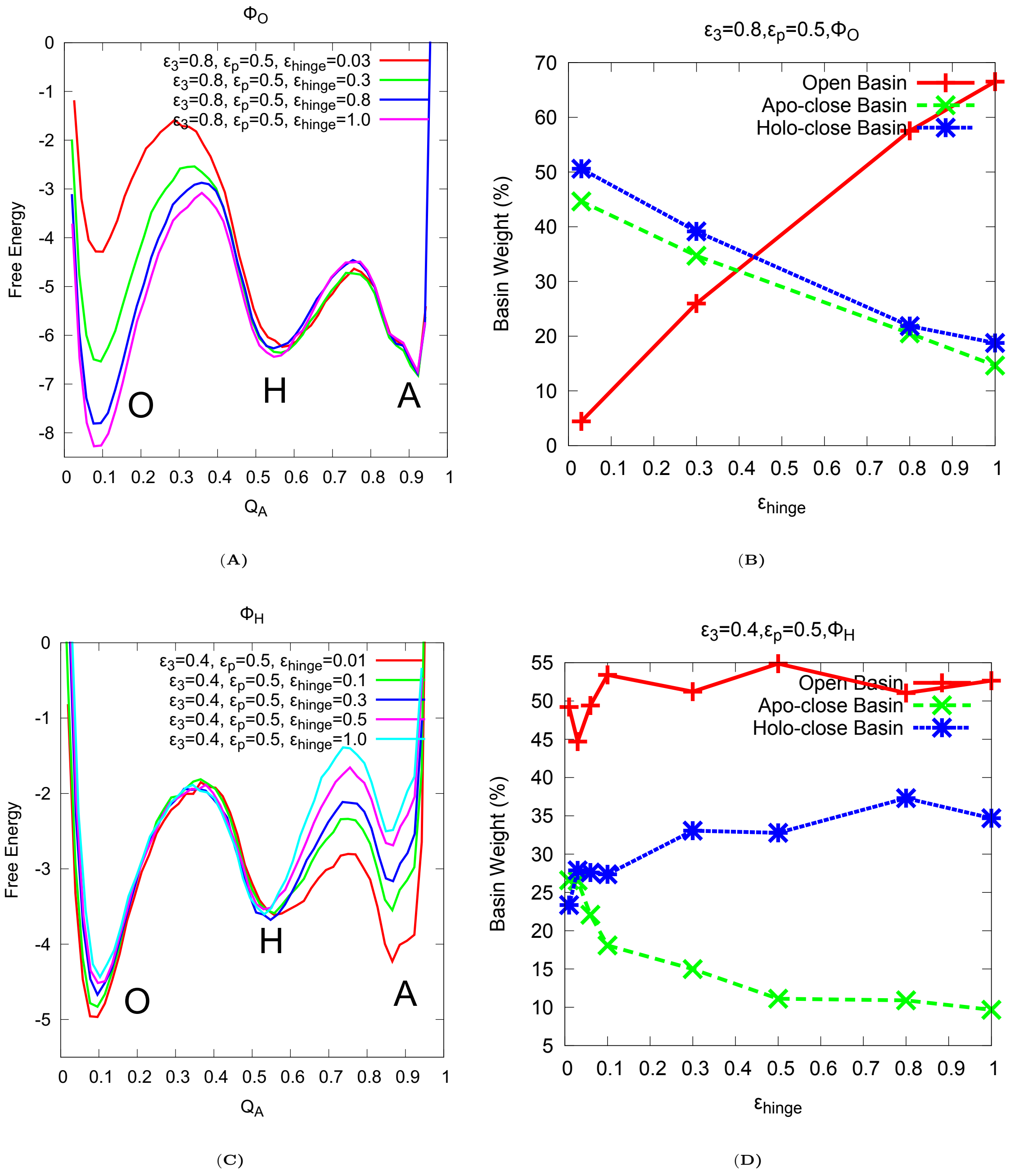

Fig. 8. Effects of ϵ_{hinge} on the thermodynamics with different native angle models. (A) and (C) show free energy profiles as a function of Q_A with different hinge flexibility with Φ_O model and Φ_H model. (B) and (D) show the correlations between basin probability, which reflects the stability for corresponding state, and hinge flexibility with Φ_O model and Φ_H model. For Φ_O model, decreasing of ϵ_{hinge} decrease the depth of open basin, for destabilizing the open-close basin and increasing the stability of apo and holo-close basin. But it has little influence on either $H \rightarrow A$ or $A \rightarrow H$ transition barrier. For Φ_H model, hinge flexibility mostly decreases the stability of holo-close basin and increases the stability of apo-close basin. However, there is little impact on $H \rightarrow O$ free energy barrier and stability of open basin. Overall, our results clearly show that the effect of hinge flexibility on protein stability. Although the models have different native biases, they all imply that increasing hinge flexibility can decrease the free energy barriers from their native basin to other basins.

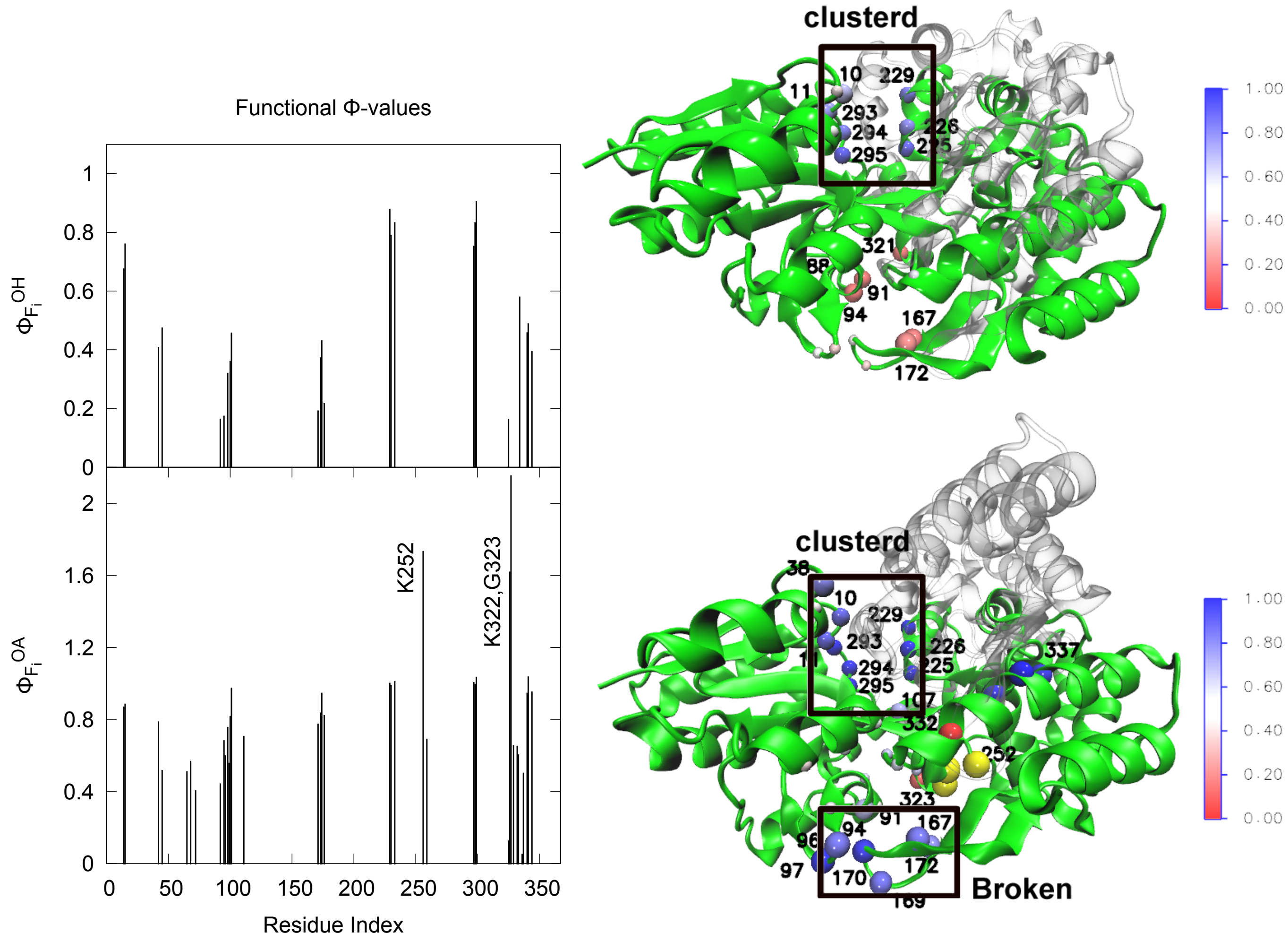

(A) (B)

Fig. 9. Functional Φ_{F_i} values analysis of transition state ensembles TS_{OH} and TS_{OA}. (A) Functional $\Phi_{F_i}^{OH}$ and $\Phi_{F_i}^{OA}$ as a function of residue index. (B) Functional $\Phi_{F_i}^{OH}$ and $\Phi_{F_i}^{OA}$ mapped onto the two-ending crystal structures. Upper subfigure shows the open and holo-close structures. Lower subfigure shows the open and apo-close structures. Open structure is represented by green cartoon. The CTD of apo- or holo-close structure is demonstrated by grey transparent cartoon. Note that the structures of open and close states are aligned according NTD. The residues with Φ_{F_i} values are represented by spheres with different colors according to the right color bar in which blue means high value and red means low value. The best targets for mutations to perturb the transition rates are those residues for which Φ_{F_i} close to either 0 or 1. Here, the residues with modest Φ_{F_i} values (0.4 to 0.6) are represented by small white spheres. In addition, if $\Phi_{F_i RP}$ is larger than 1, the residue will be colored in yellow. From the TS_{OH} analysis, it indicates that residues in NTD (D10, K11, K293, P294, L295) and these residues in CTD (P225, W226, S229) play a key role in stabilizing the holo-close state ($< Q_i >^H$ is larger than $< Q_i >^O$) and contribute to closure of MBP ($\Phi_{F_i OH} > 0.6$). In holo-close state, these residues are clustered together to form a tight interaction network which can be seen in contact probability map in Fig. 11, and thus close the tip of active site groove. Our model also predicts that residues F88, D91, R94, N96, Y167, N169, Y172 and Q321 are especially close to open state in TS_{OH} ($\Phi_{F_i OH} < 0.2$). In addition, the $\Phi_{F_i OH}$ values for residues Y95, N96, G170, K171 (white small spheres) are about 0.5 and $< Q_i >^O$ values for them are larger than $< Q_i >^H$, indicate that the interactions between the balancing loop and NTD are partially broken in TS_{OH}, however the interactions between D91, R94 and Y167, Y172 are not broken. From the TS_{OA} analysis, it indicates that the clustered residues are the same as in TS_{OH} except that K38 also plays an important role to stabilize the apo-close state. Furthermore, in helix in C2 domain, there are not only high Φ_{F_i} residues (W336, Y337, R340), but also low Φ_{F_i} residue (M332). We found $\Phi_{F_i}^{OA}$ values of K252, K322 and G323 are larger than 1 (yellow spheres) caused by that $< Q_i >^{O \cap A}$ is out of the range of $< Q_i >^O$ and $< Q_i >^A$. Indeed, for K252, $< Q_i >^O$, $< Q_i >^{O \cap A}$ and $< Q_i >^A$ are 3.8, 2.8, 3.3 respectively. For K322, they are 3.9, 2.6, 3.1. And 2.7, 1.6, 2.2 for G323. K252 is located at the loop region of linker1, and K322 and G323 are located at the loop region of linker2 (balancing linker).

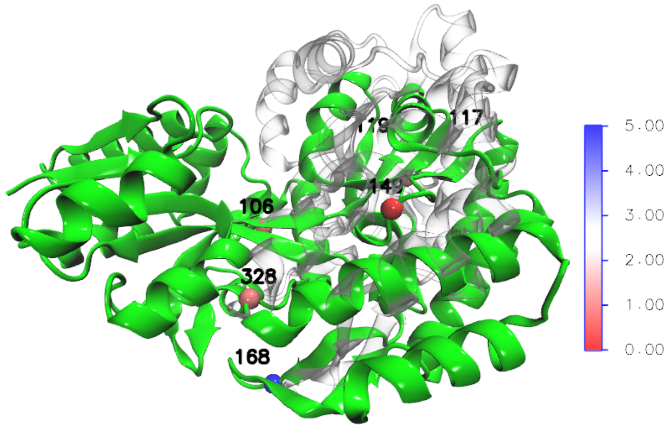

(A)

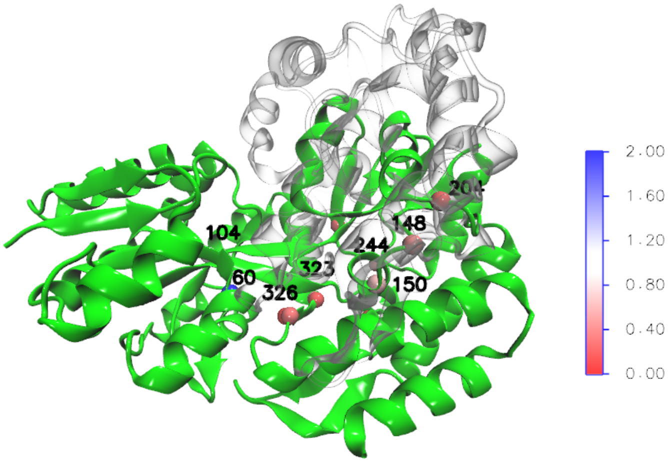

(B)

Fig. 10. Local Cracking for open-to-close transitions. (a) Unfolding points at transition state of O-H transition. (b) Unfolding points at transition state of O-A transition. Our model indicates that the local unfolding points may contain V106, L117, P119, E149, E168 and N328 in TS_{OH}. And for TS_{OA}, these unfolding region may include H60, I104, P150, Q148, T204, P244, K322, G323, M326. These residues are located at the loop regions with high flexibility with exception of I104 and V106 which are located in linker1 and approach to the known hinge residue E107. Especially, the high Ψ_i value for E168 which is located at balancing loop may be caused by the high flexibility of balancing loop due to not interacting with NTD. In addition, N328 in TS_{OH} and K322, G323, M326 in TS_{OA} are located at the loop region of balancing linker.

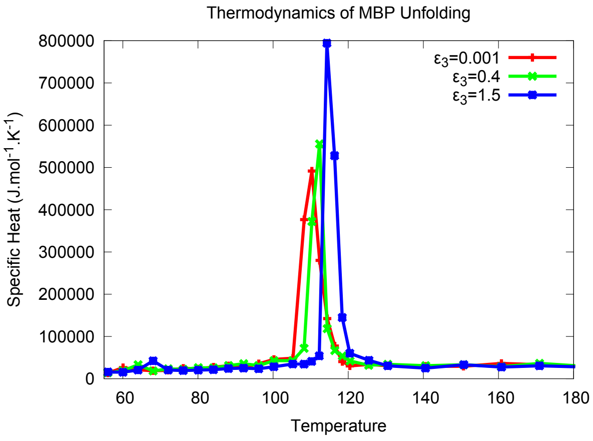

(A)

Fig. 11. Effects of ligand concentration on specific heat curves. The parameters are $\epsilon_1 = 1.0, \epsilon_2 = 0.4, \epsilon_o = 1.0, \epsilon_a = 1.3, \epsilon_h = 0.2, \epsilon_p = 0.5, \epsilon_{hinge} = 0.01$, with Φ_H. A shift of the peak of the specific heat curves to higher temperatures is observed when increasing ϵ_3. This suggests the increase of protein stability. Along with the increase of stability, the folding cooperativity increases as well. This is evident from the narrowing of the specific heat profile when increasing ϵ_3. From the results in simulations by our model it reveals that, under higher ligand concentration or with stronger ligand-binding interactions, the ligand binding links the two domains more tightly, leading to higher cooperativity, as well as stability.

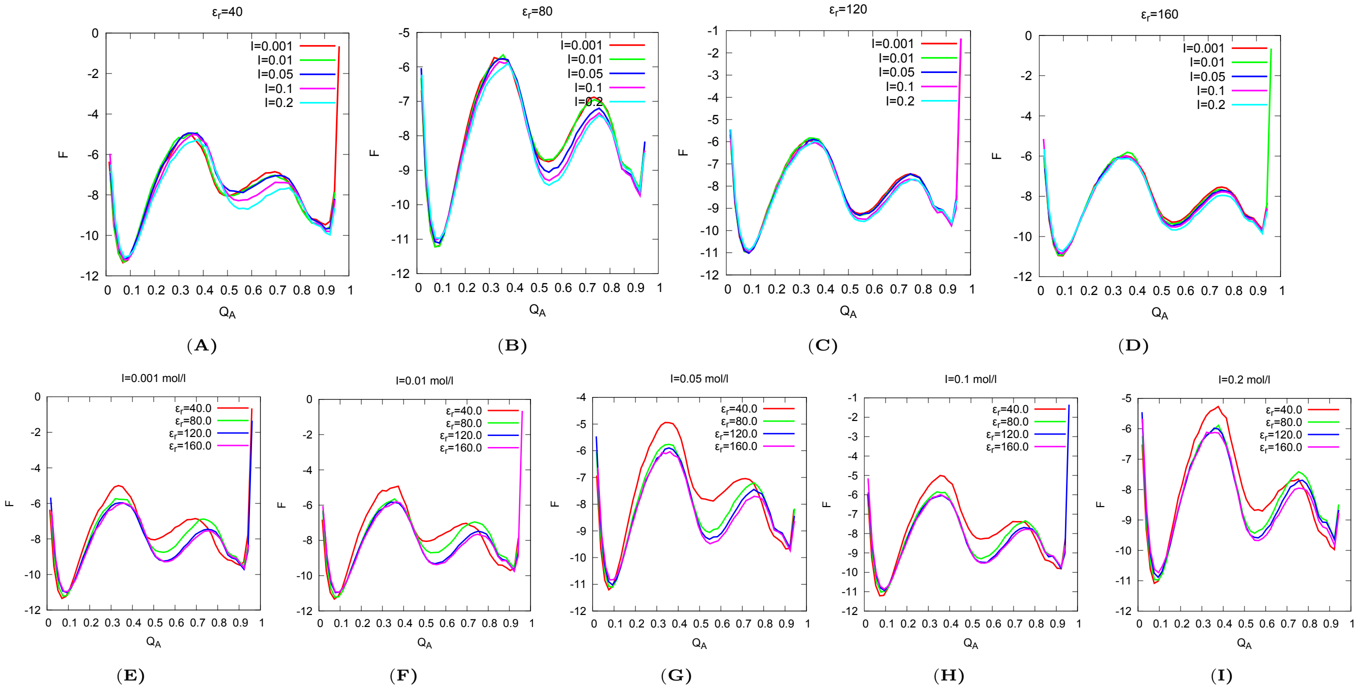

Fig. 12. Test of ϵ_r and ion strength. $\epsilon_1 = 1.0, \epsilon_2 = 0.4, \epsilon_o = 1.0, \epsilon_a = 1.4, \epsilon_h = 0.2, \epsilon_3 = 0.8$, $\epsilon_p = 0.5$, $\epsilon_{hinge} = 1.0$, with Φ_O.
